# Maternal overweight and obesity during pregnancy and sex‐dependent dysregulation of the materno‐fetal stress axis

**DOI:** 10.1002/ijgo.71165

**Published:** 2026-07-01

**Authors:** Thorsten Braun, Marie Hofmann, Rebecca Rancourt, Julia Sommer, Alexandra Kühn, Loreen Ehrlich, Thomas Ziska, Kerstin Melchior, Karen Schellong, Wolfgang Henrich, Sonja Entringer, Katherine Margaret Kennedy, Andreas Plagemann, Deborah Magdalena Sloboda

**Affiliations:** ^1^ Clinic of Obstetrics Charité—Universitätsmedizin Berlin, Corporate Member of Freie Universität Berlin, Humboldt‐Universität zu Berlin, and Berlin Institute of Health Berlin Germany; ^2^ Division of Experimental Obstetrics Charité—Universitätsmedizin Berlin, Corporate Member of Freie Universität Berlin, Humboldt‐Universität zu Berlin, and Berlin Institute of Health Berlin Germany; ^3^ Department of Psychology Charité—Universitätsmedizin Berlin, Corporate Member of Freie Universität Berlin, Humboldt‐Universität zu Berlin, and Berlin Institute of Health Berlin Germany; ^4^ Department of Biochemistry and Biomedical Sciences McMaster University Hamilton Ontario Canada; ^5^ Department of Obstetrics and Gynecology McMaster University Hamilton Ontario Canada; ^6^ Farncombe Family Digestive Health Research Institute, McMaster University Hamilton Ontario Canada

**Keywords:** cortisol, DOHaD, gestational weight gain, hypothalamic–pituitary–adrenal axis, maternal overweight, neonatal growth, placenta, placental 11βHSD2, stress

## Abstract

**Objective:**

Maternal obesity and excess gestational weight gain (GWG) are linked to adverse perinatal outcomes, potentially via disruption of the maternal–fetal stress axis. Placental HSD11B2 protects the fetus from excess glucocorticoids and may influence fetal programming during maternal metabolic stress. The present study assessed maternal overweight and GWG effects on stress axis and fetal development.

**Methods:**

We conducted a prospective study of 175 singleton pregnancies at Charité University Berlin, recruited by pre‐pregnancy body mass index (BMI: normal, overweight, obese). Maternal weight, hair and serum cortisol, neonatal anthropometrics, and placental HSD11B2 expression and methylation were analyzed during pregnancy, at delivery, and/or 6 months postpartum. Linear regression and correlation analyses were applied.

**Results:**

Excessive gestational weight gain (GWG) was more common in obese women, who also showed higher postpartum weight retention. First‐trimester hair cortisol and cortisone, reflecting chronic stress, were elevated in overweight and obese groups. While serum cortisol levels were similar, obese women showed a greater increase from the second to third trimester. Neonatal means did not differ consistently, yet macrosomia and small for gestational age rates were higher in offspring of obese mothers. Elevated maternal cortisol in late pregnancy and postpartum correlated with altered infant growth. Placental HSD11B2 was not GWG‐dependent but varied by sex and BMI. Promoter methylation was linked to infant body composition and growth.

**Conclusion:**

Maternal overweight may relate to chronic hypothalamic–pituitary–adrenal (HPA) axis activation and altered placental HSD11B2, with sex‐specific placental adaptations affecting fetal glucocorticoid exposure and offspring development. These findings support integrated perinatal strategies targeting nutrition and stress.

## 1 INTRODUCTION

Obesity and related non‐communicable diseases (NCDs) represent a critical global health issue, causing 38 million deaths annually and heavily burdening healthcare systems.^1^, ^2^ In parallel, childhood obesity is also increasing, with 15% of children aged 3–17 classified as overweight.^3^ Mounting evidence supports a developmental origins model, where early life factors, including perinatal exposures, shape long term health trajectories and contribute to obesity risk in offspring.^4^

Of the many early exposures, excessive gestational weight gain (GWG) and maternal overweight or obesity (body mass index [BMI, calculated as weight in kilograms divided by the square of height in meters] ≥ 25) are consistently identified as key predictors of childhood obesity,^5^, ^6^, ^7^, ^8^ metabolic syndrome,^9^ and intergenerational transmission of cardiometabolic disease.^10^ With high obesity rates among individuals of reproductive age these risks are amplified, linking maternal adiposity to adverse outcomes for mothers and children.^8^, ^11^, ^12^, ^13^, ^14^, ^15^ While multiple mechanisms underlie these associations, stress physiology, and in particular, maternal hypothalamic–pituitary–adrenal (HPA) axis regulation, has emerged as a potentially important contributor.

Maternal obesity is associated with altered perinatal cortisol dynamics and hypothalamic–pituitary–adrenal (HPA) axis dysregulation, which may impair maternal metabolism and appetite regulation,^16^, ^17^, ^18^, ^19^ and alter fetal and newborn development. Elevated maternal cortisol levels during pregnancy have been associated with increased infant adiposity, suggesting perinatal glucocorticoid exposure in individuals with obesity^20^, ^21^, ^22^ increase obesity risk in offspring.^23^ Proposed mechanisms include HPA axis hyperactivity or increased cortisol clearance as a compensatory adaptation.^24^ In non‐obese pregnancies, maternal cortisol secretion rises across gestation, but in obese pregnancies this rise is blunted. Pregnant individuals with obesity often show attenuated diurnal cortisol rhythms compared to lean individuals,^25^, ^26^ with lower morning cortisol, blunted cortisol awakening responses and blunted HPA responses to stress. Similar cortisol dysregulation is seen in non‐pregnant individuals with obesity; thus, perhaps elevated pre‐pregnancy BMI may contribute to maternal hypercortisolism during pregnancy. This state may expose the fetus to elevated glucocorticoids, increasing the risk of adverse development and long‐term health outcomes, including metabolic,^13^, ^14^, ^27^, ^28^, ^29^ respiratory and autoimmune conditions in children.^30^, ^31^, ^32^

However, pregnancy is not without compensatory mechanisms to protect the fetus from maternal cortisol. The placenta plays a pivotal role in regulating fetal exposure to maternal cortisol. As a critical mediator of maternal‐fetal exchange, it adapts structurally and functionally to intrauterine stressors, including maternal adiposity. One of its key protective mechanisms is the expression of the enzyme 11β‐hydroxysteroid dehydrogenase type 2 (HSD11B2), which inactivates maternal cortisol to its keto‐metabolite, cortisone, thereby buffering the fetus from excess maternal glucocorticoids.^33^ However, obesity and/or GWG may influence this enzymatic barrier, possibly through epigenetic modifications of the HSD11B2 gene.

While obesity is linked to altered HPA‐axis regulation, maternal psychosocial stress, mental health, and socioeconomic context also shape cortisol dynamics. In the present study, we therefore interpret HPA‐axis alterations in women with higher adiposity as associations that likely involve both metabolic and psychosocial pathways, rather than attributing them solely to adiposity.

The present study investigated the interplay between maternal adiposity, gestational weight gain, maternal cortisol levels and placental HSD11B2 expression with the goal of understanding how these factors contribute to altered maternal‐fetal stress physiology and adverse developmental programming.

## 2 MATERIALS AND METHODS

### 2.1 Participants

The study protocol was reviewed and approved by the Charité University ethics committee (EA 4/059/16), and all participants provided written informed consent.

In this prospective cohort study conducted at a single tertiary referral center, healthy women aged ≥18 years with singleton pregnancies were recruited between 9 and 17 weeks of gestation during routine antenatal visits as part of an ongoing research program at Charité—Universitätsmedizin Berlin, Germany. Recruitment took place at the Virchow‐Klinikum and Charité Mitte campuses between 2016 and 2020.

We excluded participants who (1) had severe chronic gastrointestinal diseases or conditions; (2) had any significant heart, kidney, liver, or pancreatic diseases; (3) had pre‐existing or gestational diabetes; (4) had depression; (5) delivered infants with severe malformations noted at birth or (6) had any signs of preterm birth and antenatal corticosteroid treatment.

### 2.2 Evaluation schedule

Participants were evaluated at five study visits: (1) at the end of the first trimester (9–17 weeks' gestation), (2) second trimester (26–28 weeks), and (3) third trimester (>33 weeks) of pregnancy, (4) at delivery, and (5) at 6 months postpartum. BMI and GWG groups were predefined exposure categories and assignment was performed from prospectively collected anthropometric data. Pre‐pregnancy body mass index (BMI) was calculated from measured height at recruitment and self‐reported pre‐pregnancy weight. BMI was then categorized into normal weight (18.5–24.9), overweight (25–29.9), and obese (30.0–39.9), according to the WHO classification criteria.^34^ At each study visit, participant weight was measured. Gestational weight gain categories were classified according to the Institute of Medicine (IOM) criteria^35^ as below, recommended or excessive gestational weight gain within BMI categories (normal maternal weight BMI 18.5–24.9 = 11.3–15.9 kg, overweight BMI 25.0–29.9 = 6.8–11.3 kg, obese BMI ≥30 = 5.0–9.1 kg).^36^ BMI categories remained fixed throughout the study; participants did not change BMI group over time. In contrast, GWG categories were evaluated longitudinally at each time point, because weight gain accrues over pregnancy. Thus, a participant's GWG classification could change over time as additional weight measurements were collected.

### 2.3 Maternal and neonatal outcome measures

An extensive set of maternal data including maternal age, weight and BMI at the beginning of pregnancy, maternal glucose and/or HbA1c during/at the end of pregnancy, weight and BMI at the end of pregnancy, maternal weight and BMI gain during pregnancy, smoking before pregnancy was collected to characterize the study cohort (Tables 1 and 2).

**TABLE 1:** General and specific maternal cohort characteristics—BMI.

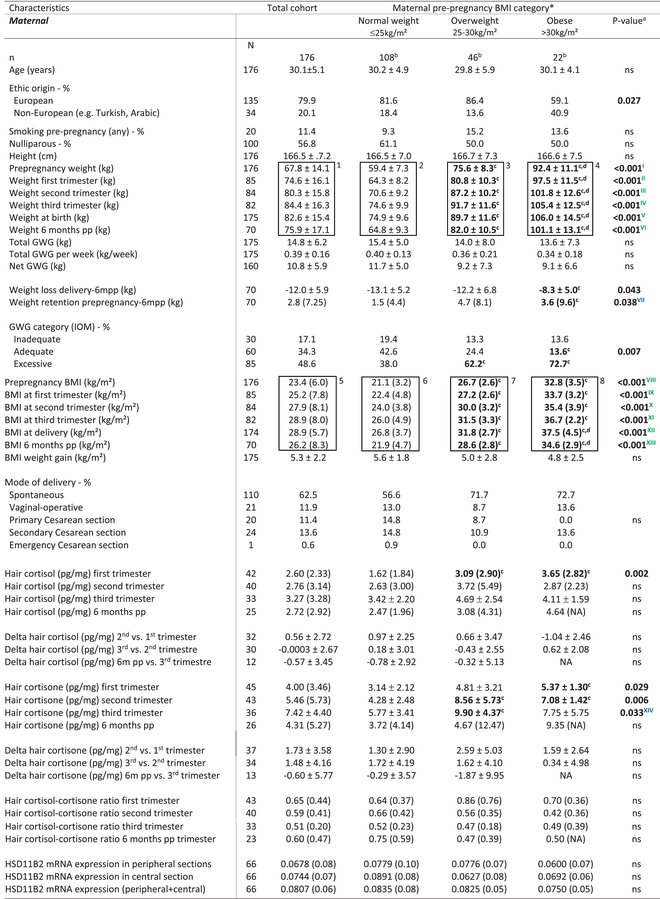
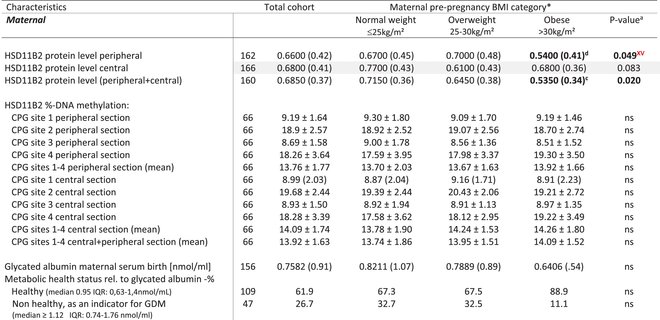

*Note*: BMI, calculated as weight in kilograms divided by the square of height in meters. Data are presented as mean (SD) and median (IQR) for normally and non‐normally distributed data, respectively or %. NET GWG = genuine maternal weight gain during pregnancy, calculated by subtracting birth weight and placental weight from total GWG. ANOVA, analysis of variance; BMI, body mass index; GDM, gestational diabetes mellitus; GWG, gestational weight gain; IOM, Institute of Medicine; IQR, interquartile range; n, total number of subjects; ns, non‐significant; N, number of subjects with characteristic; NA, not available due to low numbers; pp, postpartum; SD, standard deviation. *Normal weight: BMI ≤25, overweight BMI 25–30, obese BMI >30. ^+^Birth weight percentiles after Voigt et al.^37^ ^++^Weight and length percentile after Kromeyer‐Hauschild et al.^38^ ^+++^Centile adjusted for maternal height and for pre‐pregnancy weight (N. Rochow). ^a^Statistically significant differences (*P* < 0.05) across all pregestational BMI groups were assessed using one‐way ANOVA with Tukeyʼs post hoc test or Kruskal‐Wallis test with Dunnʼs post hoc test and Fisher exact or chi‐squared tests with Bonferroni correction, as appropriate. ^b^ *n* across variables: Normal weight = 74–89, overweight = 39–51, obese = 49–65. ^c^Statistically significant difference versus normal weight. ^d^Statistically significant difference versus overweight. Sex differences are indicated in **blue for males only**, **red for females only** and **green for males and females**, and **black of sex‐independent**. Further outcome details can be found in the Supporting Information S1.

**TABLE 2:** General and specific maternal cohort characteristics—GWG.

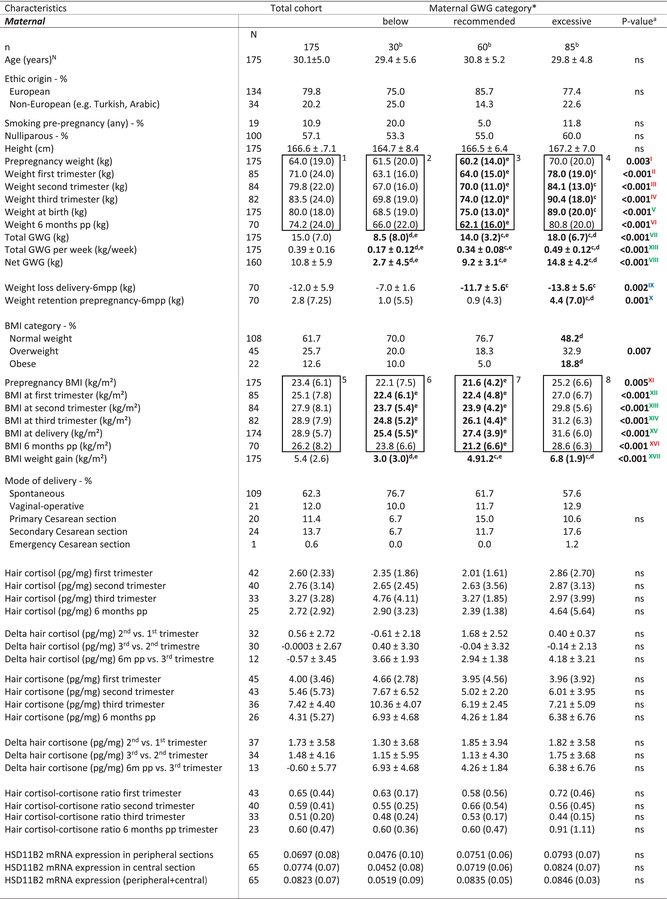
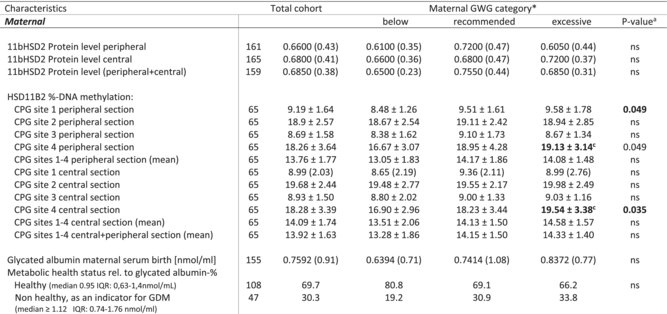

*Note*: BMI, calculated as weight in kilograms divided by the square of height in meters. Data presented as mean (SD) and median (IQR) for normally and non‐normally distributed data, respectively or %. NET GWG = genuine maternal weight gain during pregnancy, calculated by subtracting birth weight and placental weight from total GWG. ANOVA, analysis of variance; BMI, body mass index; GDM, gestational diabetes mellitus; GWG, gestational weight gain; IQR, interquartile range; IOM, Institute of Medicine; n, total number of subjects; ns, non‐significant; N, number of subjects with characteristic; pp, postpartum; SD, standard deviation. *Gestational weight gain category after IOM: normal maternal weight BMI 18.5–24.9 = 11.3–15.9 kg, overweight BMI 25.0–29.9 = 6.8–11.3 kg, obese BMI ≥30 5.0–9.1 kg;^36^ ^+^birth weight percentiles after Voigt et al.^37^ ^++^Weight and length percentile after Kromeyer‐Hauschild et al.^38^ ^+++^Centile adjusted for maternal height and for pre‐pregnancy weight (N. Rochow). ^a^Statistically significant differences (*P* < 0.05) across all pregestational BMI groups were assessed using one‐way ANOVA with Tukey's post hoc test or Kruskal‐Wallis test with Dunn's post hoc test and Fisher exact or chi‐squared tests with Bonferroni correction, as appropriate. ^b^ *n* across variables. ^c^Statistically significant difference versus below recommended weight gain. ^d^Statistically significant difference versus recommended weight gain. ^e^Statistically significant difference versus excessive weight gain. Sex differences are indicated in **blue for males only**, **red for females only** and **green for males and females**, and **black of sex‐independent**. Further outcome details can be found in Supporting Information S1.

Data on pregnancy and infant outcomes were obtained from medical charts, including fasting glucose and glucose response to 50 g challenge test, pre‐eclampsia, mode of delivery, complete sets of neonatal anthropometrics (weight, body length, head circumference), ponderal index (calculated by 100 × birth weight [kg]/body length [cm]^3^), gestational age, birth trauma including shoulder dystocia. Birth weight was categorized as small (<10%tile for weight), appropriate (50%tile) or large (>90%tile) for gestational age assessed by Voigt et al.^37^ and Charité growth curves.^39^ In addition, birth weight centiles adjusted for maternal height and for pre‐pregnancy weight were calculated.^40^, ^41^ Placenta circumference (short and long distance) was calculated. Umbilical cord blood gases with umbilical artery (UApH) and vein pH (UVpH), base excess, 1‐, 5‐ and 10‐min Apgar scores, and placental parameters (placental weight, placenta width, placenta thickness, placenta surface area) were recorded. Infant weight and length percentiles at 6 months of age were calculated according to Kromeyer et al.^38^

### 2.4 Sample collection

Maternal serum and plasma samples were collected from each participant at the visits during pregnancy and at delivery (at a stage of 4–5 cm cervical dilatation); hair samples were collected at each prenatal visit; fetal umbilical cord (arterial and venous) samples were collected immediately after delivery and cord clamping. Whole central, medial and peripheral placenta biopsies were collected according to a systematic and uniform random sampling protocol as described previously.^42^, ^43^ Tissue samples were snap frozen in liquid nitrogen and stored at −80°C until further use.

### 2.5 HPA axis biochemistry

#### 2.5.1 Hormone analysis

Maternal and fetal plasma cortisol concentrations were determined using a competitive enzyme‐linked immunosorbent assay (ELISA; Cortisol ELISA MS E‐5000, LDN Labor Diagnostika Nord GmbH, Germany), according to the manufacturer's instructions. All samples were analyzed in duplicate according to the manufacturer's instructions using standard curves ranging from 0 to 800 ng/mL. The assay sensitivity was 7.74 ng/mL. Intra‐ and interassay coefficients of variation were 5.6% and 6.5%, respectively.

#### 2.5.2 Hair analysis

Hair cortisol, cortisone, testosterone, progesterone and dehydroepiandrosterone (DHEA) concentrations were assessed using column‐switching liquid chromatography–atmospheric pressure chemical ionization–tandem mass spectrometry (LC‐APCI‐MS/MS). Analyses were performed at an external laboratory (Dresden LabService GmbH, Germany).^44^ Hair strands were cut close to the scalp and processed according to standardized protocols.^45^ The proximal 3 cm of hair were analyzed to reflect cumulative glucocorticoid exposure over approximately three months, based on an average hair growth rate of 1 cm/month.

#### 2.5.3 Quantitative PCR

Genomic DNA/RNA were extracted from frozen human placental tissue using the Zymo Quick‐DNA/RNA Isolation Miniprep Plus Kit (cat. no.: D7003, Zymo Research), following the manufacturer's two‐step protocol to ensure efficient separation. A total of 66 representative placentas were analyzed, with one central and one peripheral sample obtained from each placenta (*n* = 132). Tissue samples were mechanically disrupted and homogenized under standardized conditions. Nucleic acid concentration and purity (A260/A280 ratios) were measured in duplicate using a FLUOstar Omega microplate reader (BMG Labtech). RNA was reverse transcribed into complementary DNA (cDNA) using the iScript cDNA Synthesis Kit (Bio‐Rad, cat. no.: 1708881). Commercially available TaqMan probe‐based gene expression assays were used (Life Technologies, Carlsbad, California, USA) and were run on an Applied Biosystems 7500 instrument according to the manufacturer's protocol (Applied Biosystems, Waltham, Massachusetts, USA). Expression levels were normalized to the housekeeping gene RPL‐19 and PPIA according to previous validation studies.^46^ When possible, exon‐spanning primer sets were selected, qPCR was performed as duplex qPCR with housekeeping genes. Assays were carried out in triplicate, and relative gene expression was calculated using the 2^−ΔCT^ method corrected for the amplification efficiency calculated from standard curves for all primer sets.^47^ TaqMan gene expression assays: Hsd11b2: Hs00388669_m1, RPL‐19: Hs02338565_gH and PPIA: Hs99999904_m1.

#### 2.5.4 DNA methylation

Genomic DNA extracted from placental tissue was bisulfite‐converted using the EZ DNA Methylation Kit (Zymo Research), according to the manufacturer's instructions. Pyrosequencing was conducted to quantify DNA methylation levels at defined CpG sites within the HSD11B2 promoter region (Figure 1). Primer sequences were based on previously validated assays.^48^ PCR amplification and sequencing were carried out in triplicate. Methylation levels were expressed as the percentage of methylated cytosines at each individual CpG site (Figure 2).

**FIGURE 1:**
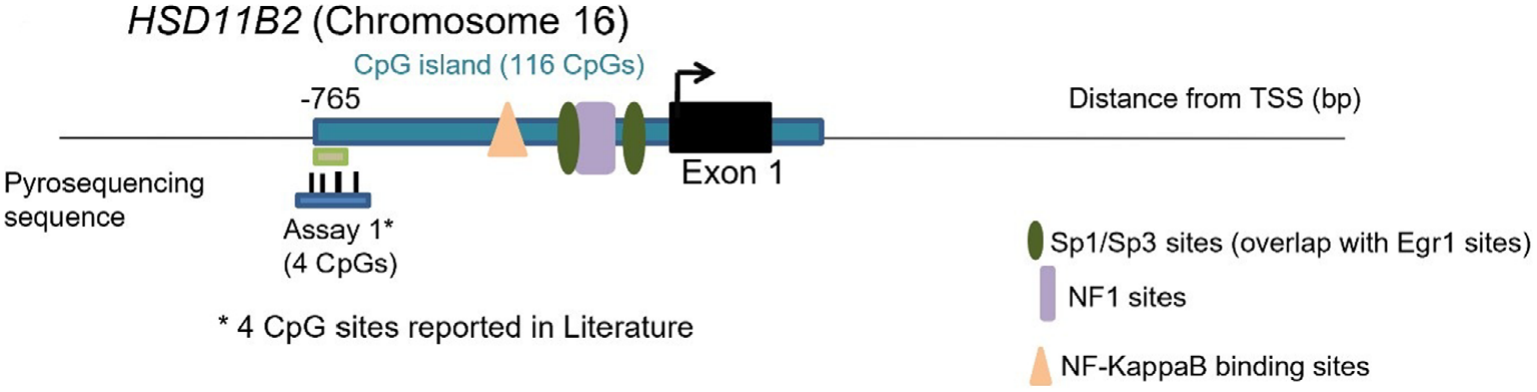
DNA methylation analysis in the HSD11B2 assay region. Schematic illustration of the DNA methylation assay for the promoter region of HSD11B2 within the CpG island including predicted transcription factor binding site FOXO1. *Zhao et al. reported correlation of methylation at specific CpG sites inversely associated with fetal growth measures (birth weight, ponderal index).^48^

**FIGURE 2:**
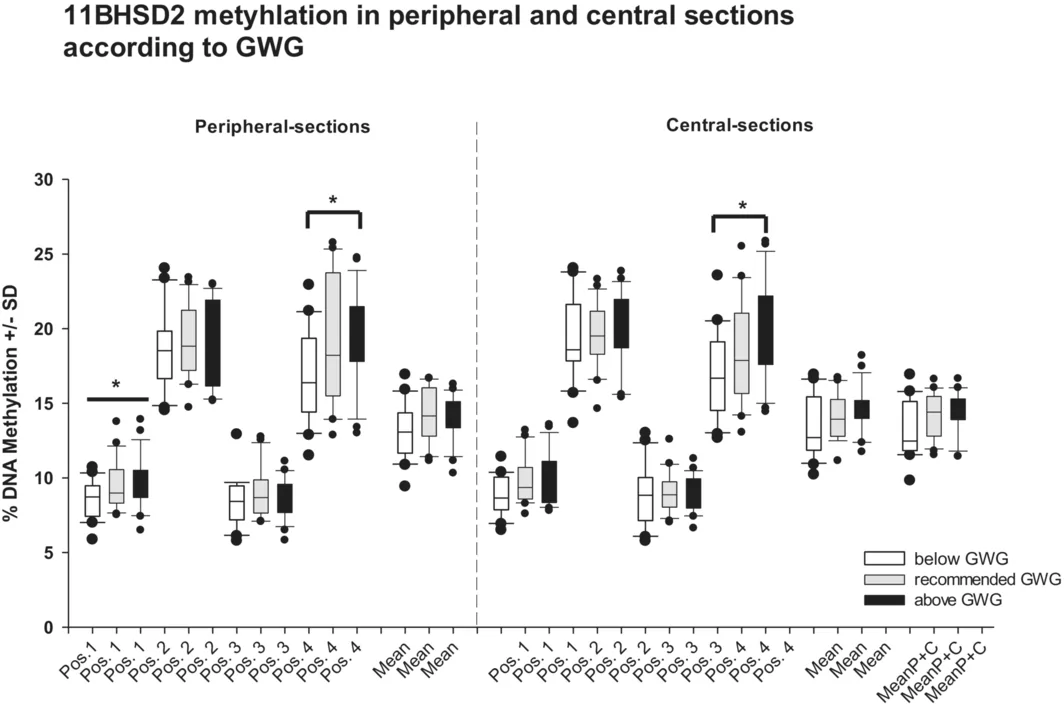
Percent DNA methylation at each CpG site and mean in the HSD11B2 assay according to gestational weight gain (GWG) for placenta sections peripheral and central (below GWG; white; *n* = 20) (recommended GWG, gray; *n* = 21) (above GWG, black; *n* = 24). Data are means ± SD.

#### 2.5.5 Western blotting

Placental protein expression of HSD11B2 was quantified by Western blotting. Frozen placental tissue was lysed, and equal amounts of protein (20 μg) were separated via SDS‐PAGE and transferred to PVDF membranes. Membranes were probed with a sheep anti‐human HSD11B2 primary antibody (1:8000, MAB8630 R&D Systems, Wiesbaden, Germany), followed by horseradish peroxidase‐conjugated secondary antibodies (1:2000, HAF0118, R&D Systems, Wiesbaden, Germany). Detection was performed using chemiluminescence, and band intensities were normalized to total protein using Stain‐Free imaging technology (Bio‐Rad). All experiments included an internal control and the HSD11B2 antibody was validated by immunohistochemical localization as previously reported.^49^

### 2.6 Statistical analysis

Maternal pre‐pregnancy BMI was calculated using the standard formula: BMI = weight (kg)/height (m).^2^ Gestational weight gain was calculated by subtracting pre‐pregnancy weight from the weight recorded at each respective visit. Net weight gain was calculated by subtracting birth weight and placental weight from total GWG. Laboratory personnel assessing hormonal and placental outcomes were blinded to clinical group status where feasible (e.g., samples labeled with study IDs only), and statistical analyses were stratified by BMI and/or GWG group as prespecified in the analysis plan. Descriptive statistics are presented as means ± standard deviations (SD) or as counts and percentages, as appropriate. All continuous variables were tested for normal distribution using the Kolmogorov–Smirnov test and an evaluation of distribution plots. Group comparisons were analyzed using one‐way analysis of variance (ANOVA) followed by Tukey's post hoc test, or the Kruskal‐Wallis test followed by Dunn's post hoc test, along with Fisher exact test or Chi‐square tests as appropriate. Further outcome details can be found in the Supporting Information S1. Spearman's correlation coefficients (r) and/or linear regression models (unstandardized B coefficients with 95% confidence intervals [95% CI]) were calculated to assess relationships between maternal anthropometric/HPA parameters and neonatal and infant anthropometric outcomes, as well as cord‐blood hormone levels. For regression analyses, normal distribution for respective variables were achieved through transformation using the Box‐Cox method.^50^

Categorical variables were dichotomized as required. The following parameters were examined as independent variables for their association with birth outcomes: anthropometry, which includes maternal height, pre‐pregnancy weight and BMI, maternal weight at birth, BMI at birth, total BMI, GWG, and net GWG. The birth outcomes assessed were placental weight, birth weight, head circumference, length and ponderal index. All regression analyses are presented as both crude and adjusted models. Model I comprised general parameters such as maternal age, gestational age in completed weeks, parity, smoking during pregnancy, and ethnic origin. To evaluate the independent associations of maternal anthropometry with birth outcomes, further adjustment models were utilized. Specifically, for maternal anthropometry, Model II included all parameters from Model I, in addition to the infant's sex and a classification for macrosomia, defined as birth weight greater than 4000 g.

In addition, forward stepwise regression analyses were carried out to identify categorical predictors of anthropometric/hormonal birth outcomes, considering maternal parameters. In line with previous studies from our group,^51^ adjusted semipartial correlation coefficients (Sr2) were calculated for each individual (potential) predictor entered into the final models. These coefficients estimate the contribution of each factor to the total variability of the outcome variable.

In general, missing data analysis according to Little's MCAR test was performed and indicated random distribution. A *P* value less than 0.05 was considered statistically significant (two‐tailed). All calculations were performed using SPSS (version 30.0; IBM Co., Armonk, New York, USA).

## 3 RESULTS

A total of 175 participants met the inclusion criteria, consented and were included in the study.

### 3.1 Maternal anthropometrics and weight trajectories (Tables 1 and 2)

Differences in maternal BMI, corresponding to the predefined BMI categories, were consistently observed throughout pregnancy—including in the first, second, and third trimesters as well as at delivery (all *P* < 0.001) and six months postpartum. Although total GWG did not differ significantly between BMI groups, the net gestational weight gain (i.e., gain after subtracting birth weight and placental weight) was lower in obese participants compared to both normal weight and overweight participants (*P* = 0.033). Excessive GWG was most frequent among obese participants (72.2%, *P* = 0.007) (Figure 3). Participants categorized as obese were significantly more likely to gain excessive weight and significantly less likely to gain the recommended amount of weight. At 6 months postpartum, weight retention was significantly higher in participants that were categorized as obese compared with normal weight participants (*P* = 0.038). This effect was independent of parity.

**FIGURE 3:**
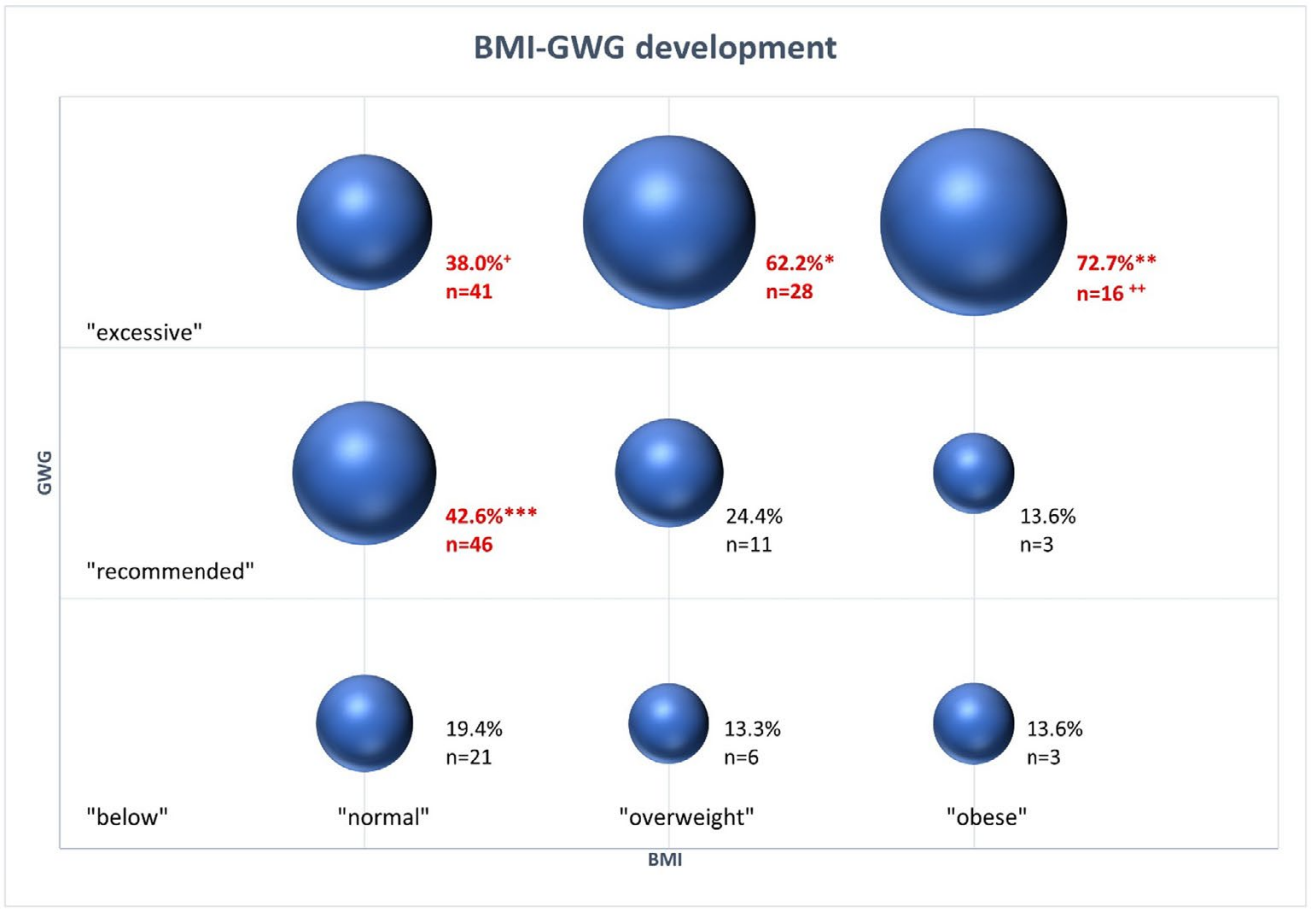
BMI‐GWG development. Participants with higher pregestational body mass index (BMI) were more likely to gain excessive weight (GWG). Despite this, up to 38% of participants with normal pregestational BMI still gained excessive weight. Significant changes were indicated as follows: ***P < 0.001, **P = 0.004, *P < 0.001, ^++^ *P* < 0.001 and ^+^ *P* = 0.004.

Differences in GWG, corresponding to the predefined GWG groups (Table 2), were consistently observed throughout pregnancy—including the first, second, and third trimesters as well as at delivery (all *P* < 0.001). At six months postpartum, participants that gained excessive GWG, retained significantly more weight, approximately 3.4 kg, compared to those in the other groups (*P* = 0.001). Multiparous participants demonstrated significantly higher weight retention compared to primiparous participants (almost double: primiparous 4.4 ± 4.9 vs. multiparous 8.3 ± 5.7 kg).

### 3.2 Anthropometry—Neonates and 6‐month‐old infants (Table 3)

**TABLE 3:** General and specific newborn cohort characteristics—BMI.

Characteristics	Total cohort		Maternal pre‐pregnancy BMI category*			*P* value
			Normal weight	Overweight	Obese	
			≤25	25–30	>30	
***Newborn***	*N*					
*n*		176	108^b^	46^b^	22^b^	
Female sex—%	75	42.6	43.5	37.0	50.0	ns
Gestational age at delivery (completed weeks)	176	40.0 (3.0)	40.0 (3.0)	39.5 (3.0)	40.0 (2.0)	ns
Preterm birth (<37 weeks)—%	24	13.6	13.9	15.2	9.1	ns
Placental weight (g)	161	570 (135)	570 (128)	555 (164)	550 (188)	ns
Placental circumference short distance (cm)	160	17.7 (2.8)	17.0 (2.0)	**18.0 (2.0)** ^**c**^	17.0 (3.1)	**0.013** ^**I**^
Placental circumference long distance (cm)	160	20.0 (2.4)	20.0 (2.4)	20.0 (3.0)	20.0 (5.0)	ns
Birth weight (g)	176	3287.4 ± 498.4	3268.9 ± 477.0	3301.2 ± 502.1	3348.9 ± 603.6	ns
Birth weight percentile^+^	176	47.3 ± 23.8	46.8 ± 22.7	49.5 ± 23.5	45.0 ± 29.8	ns
Birth weight percentile adjusted for BMI^+++^	176	51.5 (42.4)	55.9 (37.4)	44.8 (40.3)	41.4 (57.0)	ns
Birth length (cm)	176	51.0 (4.0)	51.0 (3.0)	51.0 (5.0)	51.5 (4.3)	ns
Birth length percentile^+^	176	42.5 (46.0)	41.5 (43.0)	48.0 (50.0)	37.5 (59.0)	ns
Birth length percentile adjusted for BMI^+++^	176	46.9 (49.7)	47.2 (42.9)	46.6 (54.3)	39.0 (68.3)	ns
Relative birth weight (g/cm)	176	64.7 ± 7.5	64.4 ± 7.2	65.2 ± 7.6	65.3 ± 8.6	ns
Ponderal index (g/cm^3^ × 100)	176	2.50 (0.35)	2.54 (0.34)	2.50 (0.46)	2.49 (0.25)	ns
Ratio birth weight/placental weight	158	5.75 (1.43)	5.66 (1.41)	5.70 (1.52)	6.32 (1.12)	ns
Birth weight category—%^+^						
SGA	8	4.5	1.9	2.2	**22.7** ^**c**^	
AGA	163	92.6	94.4	97.8	**72.7** ^**c**^	**0.002**
LGA	5	2.8	3.7	0	4.5	
Macrosomia (≥4000 g)—%	10	5.7	3.7	4.3	**18.2** ^**d**^	**0.038**
Head circumference (cm)	175	35.0 (2.0)	34.5 (2.5)	35.0 (2.0)	34.7 (1.3)	ns
Head circumference percentile^+^	175	49.0 (44.0)	54.0 (46.0)	47.0 (38.0)	37.0 (45.0)	ns
Head circumference percentile adjusted for BMI^+++^	175	49.9 (48.4)	60.0 (46.5)	44.7 (32.4)	32.8 (50.7)	ns
APGAR						
1‐min	173	8.8 ± 0.9	8.8 ± 0.9	8.7 ± 0.9	8.6 ± 1.0	ns
5‐min	173	9.6 ± 0.8	9.6 ± 0.9	9.6 ± 0.8	9.6 ± 0.7	ns
10‐min	173	9.8 ± 0.6	9.8 ± 0.7	9.9 ± 0.5	**9.6 ± 0.7** ^**d**^	**0.04**
Cord artery pH	175	7.26 ± 0.07	7.26 ± 0.07	7.26 ± 0.07	7.27 ± 0.07	ns
Cord base excess artery	171	−3.0 (4.3)	−2.6 (4.3)	−3.4 (4.4)	−3.0 (3.4)	ns
Cord lactate artery (mg/dL)	145	24.0 (35.2)	22.0 (36.0)	15.0 (32.8)	29.0 (26.0)	ns
Cord venous pH	152	7.35 ± 0.06	7.35 ± 0.06	7.36 ± 0.07	7.35 ± 0.05	ns
Cord lactate venous (mg/dL)	138	21.0 (30.5)	18.0 (24.6)	19.5 (32.4)	28.0 (15.0)	ns
Cord‐blood plasma levels						
Cord cortisol venous (ng/mL)	111	305.6 (154.8)	278.7 (175.9)	332.9 (146.8)	305.6 (155.0)	ns

Characteristics	Total cohort		Maternal pre‐pregnancy BMI category*			
			Normal weight	Overweight	Obese	*P* value
			≤25	25–30	>30	
***Child***						
Child weight 6 months pp (g)	82	7954 ± 1027	7836 ± 956	8048 ± 817	8183 ± 1450	ns
Child weight percentile 6 months pp^++^	82	65.0 (39.0)	61.5 (38.0)	66.0 (33.0)	76.0 (61.0)	ns ^(0.053)^
Child height 6 months pp (cm)	82	68.5 (3.0)	68.1 (4.0)	68.5 (3.0)	69.0 (2.0)	ns
Child height percentile 6 months pp^++^	82	64.0 (51.0)	64.0 (48.0)	64.0 (48.0)	76.0 (31.0)	ns
Child head circumference 6 months pp (cm)	79	43.5 ± 1.4	43.5 ± 1.4	43.7 ± 1.4	43.4 ± 1.7	ns
Child head circumference percentile 6 months pp^++^	79	58.0 (50.0)	59.5 (50.0)	58.0 (58.0)	63.0 (51.0)	ns
Child BMI 6 months pp	82	16.9 ± 1.5	16.7 ± 1.2	17.1 ± 1.5	17.2 ± 2.3	ns^**II**^
Child BMI 6 months pp percentile^++^	82	56.5 (40.0)	57.0 (27.0)	56.0 (51.0)	73.0 (71.0)	ns
BMI weight category 6 months pp^++^—%						
Underweight	5	6.1	4.3	4.8	13.3	ns
Normal weight	65	79.3	89.1	71.4	60.0	ns
Overweight	12	14.6	6.5	23.8	26.7	ns
Ponderal index 6 months pp	82	2.5 ± 0.2	2.5 ± 0.2	2.5 ± 0.3	2.5 ± 0.4	ns
Child weight gain 6 months pp (g)	82	4498 ± 887	4375 ± 888	4610 ± 603	4714 ± 1174	ns

*Note*: BMI, calculated as weight in kilograms divided by the square of height in meters. Data presented as mean (SD) and median (IQR) for normally and non‐normally distributed data, respectively or %. AGA, appropriate for gestational age; ANOVA, analysis of variance; BMI, body mass index; GWG, gestational weight gain; IQR, interquartile range; LGA, large for gestational age; n, total number of subjects; ns, non‐significant; N, number of subjects with characteristic; NA, not available due to low numbers; pp, postpartum; SD, standard deviation; SGA, small for gestational age. *Normal weight: BMI ≤ 25, overweight BMI 25–30, obese BMI >30. ^+^Birth weight percentiles after Voigt et al.^37^ ^++^Weight and length percentile after Kromeyer‐Hauschild et al.^38^ ^+++^Centile adjusted for maternal height and for pre‐pregnancy weight.^40^, ^41^ ^a^Statistically significant differences (*P* < 0.05) across all pregestational BMI groups were assessed using one‐way ANOVA with Tukey's post hoc test or Kruskal‐Wallis test with Dunn's post hoc test and Fisher exact or chi‐squared tests with Bonferroni correction, as appropriate. ^b^ *n* across variables: Normal weight = 74–89, overweight = 39–51, obese = 49–65. ^c^Statistically significant difference versus normal weight. ^d^Statistically significant difference versus overweight. Sex differences are indicated in **blue for males only**, **red for females only** and **green for males and females**, and **black of sex‐independent**. ^1^When analyzed for sex, placental circumference short distance in males was significantly lower in BMI <25 versus BMI 25–30 (*P =* 0.017) (Games‐Howell post‐hoc). ^II^When analyzed for sex, child BMI 6 months pp was significantly lower in males in BMI < 25 versus BMI > 30 (*P* = 0.008) (Games‐Howell post hoc).

Birth weight, length, head circumference, and their corresponding percentiles did not differ significantly by maternal BMI (Table 3). However, the distribution of birth weight categories revealed that the rate of macrosomia (≥4000 g) was significantly higher in neonates born to participants categorized as obese (18.2%) compared with overweight participants (4.3%; *P* = 0.038). The proportion of small for gestational age (SGA) was also higher in the obese group accompanied by a lower rate of appropriate‐for‐gestational‐age births (AGA) (*P* = 0.002), suggesting that higher maternal BMI may be linked to altered fetal growth patterns. Birth weight in participants that gained excessive GWG (Table 4) was higher than in participants that gained below the recommended amount.

**TABLE 4:** General and specific newborn cohort characteristics—GWG.

Characteristics	Total cohort		Maternal GWG category*			*P* value^a^
			Below	Recommended	Excessive	
***Newborn***						
	*N*					
*n*		175	30^b^	60^b^	85^b^	
Female sex—%	74	42.3	46.7	41.7	41.2	ns
Gestational age at delivery (completed weeks)	175	40.0 (3.0)	**39.0 (4.0)** ^**e**^	39.5 (2.0)	40.0 (2.0)	**0.038** ^**I**^
Preterm birth (<37 weeks)—%	24	13.7	**30.0**	13.3	8.2	**0.015**
Placental weight (g)	160	568 ± 119	548.5 ± 104.2	570.5 ± 136.3	573.8 ± 110.7	ns
Placental circumference short distance (cm)	159	18.0 (3.0)	17.0 (1.8)	17.5 (2.0)	18.0 (3.0)	ns
Placental circumference long distance (cm)	159	20.0 (2.0)	19.0 (2.8)	20.0 (3.0)	20.0 (3.0)	ns
Birth weight (g)	175	3340 (620)	**3064.7 ± 481.3** ^**e**^	3309.4 ± 491.7	3358.5 ± 488.8	**0.019** ^**II**^
Birth weight percentile^+^	175	47.0 (33.0)	47.5 (29.0)	50.5 (33.0)	44.0 (39.0)	ns
Birth weight percentile BMI adjusted^+++^	175	51.5 (42.4)	52.6 (35.7)	54.3 (40.0)	50.0 (43.2)	ns
Birth length (cm)	175	51.0 (4.0)	49.5 (3.3)	51.0 (4.0)	51.0 (4.0)	ns^**III**^
Birth length percentile^+^	175	43.0 (45.0)	40.0 (49.0)	42.5 (42.0)	45.0 (48.0)	ns
Birth length percentile BMI adjusted^+++^	175	46.9 (49.6)	54.3 (56.7)	48.7 (41.4)	43.4 (51.4)	ns
Relative birth weight (g/cm)	175	64.8 ± 7.46	**61.5 ± 7.34** ^**e**^	64.9 ± 7.17	65.8 ± 7.46	**0.022** ^**IV**^
Ponderal index (g/cm^3^ × 100)	175	2.50 (0.34)	2.50 (0.29)	2.52 (0.37)	2.50 (0.36)	ns
Ratio birth weight/placental weight	160	5.78 (1.43)	5.61 (1.40)	5.88 (1.51)	5.81 (1.35)	ns
Birth weight category—%^+^						
SGA	8	4.6	3.3	5.0	4.7	
AGA	162	92.6	96.7	91.7	91.8	ns
LGA	5	2.9	0.0	3.3	3.5	
Macrosomia (≥4000 g)—%	10	5.7	0.0	5.0	8.2	ns
Head circumference (cm)	174	35.0 (2.0)	34.0 (2.3)	35.0 (2.5)	35.0 (2.0)	ns^**V**^
Head circumference percentile^+^	174	49.5 (43.0)	49.0 (39.0)	50.5 (43.0)	51.0 (47.0)	ns
Head circumference percentile BMI adjusted^+++^	174	50.1 (47.0)	50.4 (48.6)	49.8 (46.3)	50.2 (47.6)	ns
APGAR						
1‐min	174	9.0 (0.0)	9.0 (0.0)	9.0 (0.0)	9.0 (0.0)	ns
5‐min	174	10.0 (1.0)	10.0 (0.0)	10.0 (0.0)	10.0 (0.0)	ns
10‐min	174	10.0 (0.0)	10.0 (0.0)	10.0 (0.0)	10.0 (0.0)	ns
Cord artery pH	174	7.26 ± 0.07	7.29 ± 0.09	7.26 ± 0.07	7.25 ± 0.07	ns
Cord base excess artery	170	−2.95 (4.3)	−2.3 (4.9)	−3.15 (4.1)	−3.10 (5.0)	ns
Cord lactate artery (mg/dL)	144	24.0 (35.3)	24.0 (36.5)	17.0 (41.3)	26.0 (32.6)	ns
Cord venous pH	151	7.35 ± 0.7	7.39 (0.09)	7.35 (0.06)	**7.34 (0.06)** ^**c**^	**0.021**
Cord lactate venous (mg/dL)	137	21.0 (30.7)	22.0 (33.2)	13.0 (33.7)	22.0 (27.6)	ns
Cord‐blood plasma levels						
Cortisol venous (ng/mL)	111	305.6 (154.8)	236.3 (205.1)	342.1 (162.5)	289.8 (146.9)	ns

Characteristics	Total cohort		Maternal GWG category*			*P* value^a^
			Below	Recommended	Excessive	
***Child***						
Child weight 6 months pp (g)	82	7954 ± 1027	7614 ± 999	8036 ± 899	8018 ± 1096	ns
Child weight percentile 6 months pp^++^	82	65.0 (39.0)	48.9 ± 27.6	63.6 ± 23.7	61.4 ± 26.9	ns
Child height 6 months pp (cm)	82	68.5 (3.0)	67.5 (3.0)	69.0 (4.0)	68.3 (4.0)	ns
Child height percentile 6 months pp^++^	82	64.0 (51.0)	62.5 (41.0)	76.0 (31.0)	64.0 (48.0)	ns
Child head circumference 6 months pp (cm)	79	43.5 ± 1.4	43.4 ± 1.74	43.8 ± 1.49	43.5 ± 1.31	ns
Child head circumference percentile 6 months pp^++^	79	58.0 (50.0)	39.5 (55.0)	70.5 (28.0)	58.0 (60.0)	ns
Child BMI 6 months pp	82	16.8 (1.6)	16.8 ± 1.64	16.6 ± 1.25	17.1 ± 1.64	ns
Child BMI 6 months pp percentile^++^	82	56.5 (40.0)	51.7 ± 31.1	53.4 ± 26.5	59.0 ± 30.0	ns
BMI weight category 6 months pp^++^—%						
Underweight	5	6.1	0.0	8.7	6.7	
Normal weight	65	79.3	85.7	82.6	75.6	ns
Overweight	12	14.6	14.3	8.7	17.8	
Ponderal index 6 months pp	82	2.4 (0.26)	2.39 (0.43)	2.42 (0.28)	2.49 (0.22)	ns
Child weight gain 6 months pp (g)	82	4498 ± 887	4.353 ± 950	4397 ± 752	4594 ± 936	ns

*Note*: BMI, calculated as weight in kilograms divided by the square of height in meters. Data presented as mean (SD) and median (IQR) for normally and non‐normally distributed data, respectively or %. AGA, appropriate for gestational age; ANOVA, analysis of variance; BMI, body mass index; GWG, gestational weight gain; IOM, Institute of Medicine; IQR, interquartile range; LGA, large for gestational age; n, total number of subjects; ns, non‐significant; N, number of subjects with characteristic; pp, postpartum; SD, standard deviation; SGA, small for gestational age. *Gestational weight gain category after IOM: normal maternal weight BMI 18.5–24.9 = 11.3–15.9 kg, overweight BMI 25.0–29.9 = 6.8–11.3 kg, obese BMI ≥30 = 5.0–9.1 kg. ^+^Birth weight percentiles after Voigt et al.^37^ ^++^Weight and length percentile after Kromeyer‐Hauschild et al.^38^ ^+++^Centile adjusted for maternal height and for pre‐pregnancy weight after Voigt et al.^40^, ^41^ ^a^Statistically significant differences (*P* < 0.05) across all pregestational BMI groups were assessed using one‐way ANOVA with Tukey's post hoc test or Kruskal‐Wallis test with Dunn's post hoc test and Fisher exact or chi‐squared tests with Bonferroni correction, as appropriate. ^b^ *n* across variables. ^c^Statistically significant difference versus below recommended weight gain. ^d^Statistically significant difference versus recommended weight gain. ^e^Statistically significant difference versus excessive weight gain. Sex differences are indicated in blue for males only, red for females only and green for males and females, and black of sex‐independent. Further outcome details can be found in the Supporting Information S1.

### 3.3 Maternal endocrine profiles (Tables 1 and 2)

Although absolute maternal serum cortisol levels in the first, second, and third trimesters were not significantly different by BMI, the change in cortisol from the second to the third trimester was significantly greater in participants categorized as obese compared with normal‐weight participants (*P* = 0.041; Figure 4b).

**FIGURE 4:**
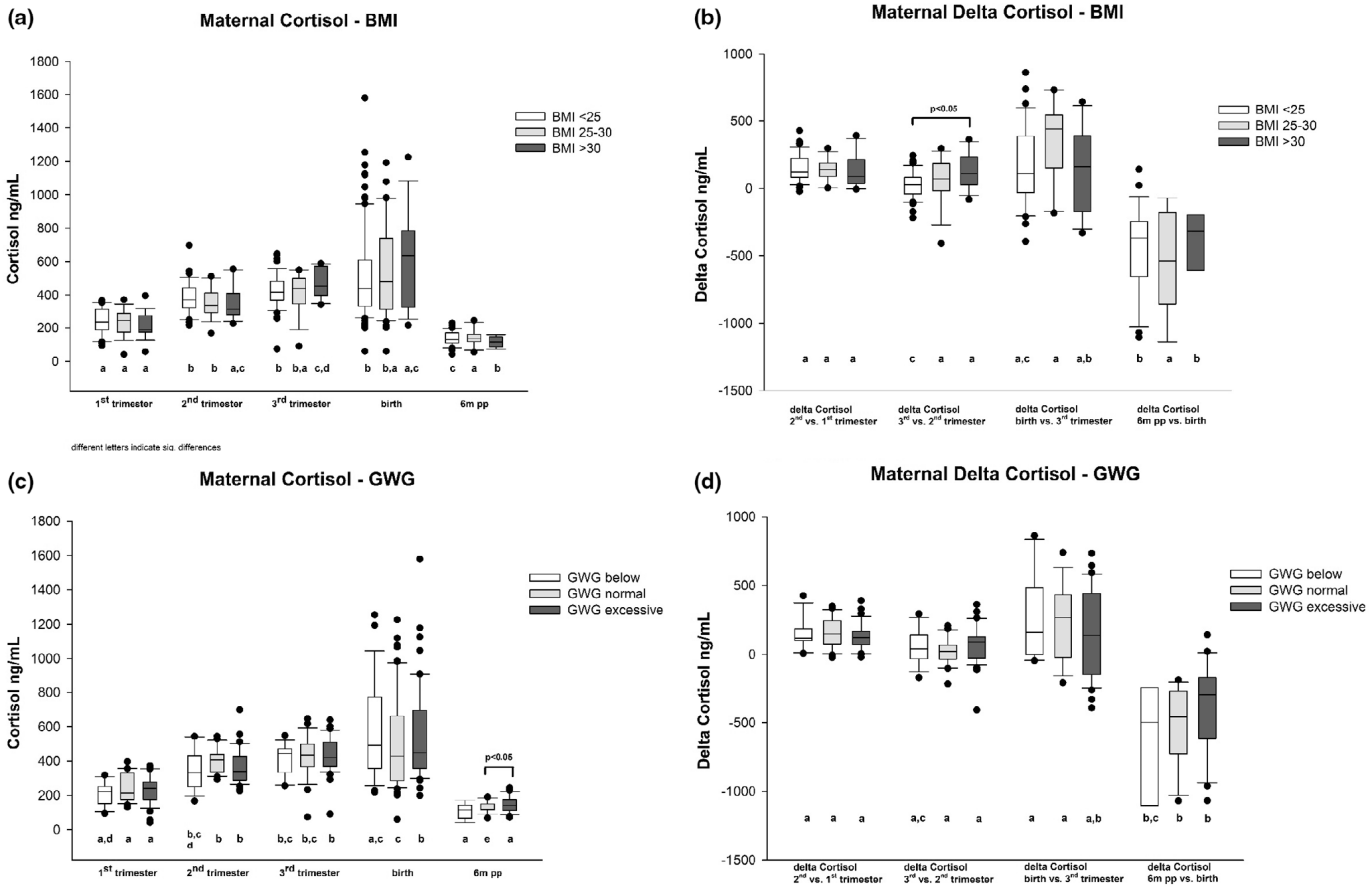
Maternal cortisol and delta cortisol levels according to pregestational body mass index (BMI) and gestational weight gain (GWG). Different letters indicate significant differences with *P* < 0.05.

Hair cortisol levels were used in the first trimester as a marker for chronic maternal stress over the last three months (Table 1) and showed significantly higher levels in overweight and obese participants compared to the normal weight participants (*P* = 0.002). Obese participants had higher levels of hair cortisone, in the first trimester compared to the normal weight group (*P* = 0.029). In the second trimester, both overweight and obese participants had significantly higher hair cortisone levels than normal weight participants (*P* = 0.006). Because hair cortisol integrates secretion over roughly three months, these findings suggest chronic differences in HPA‐axis activity across BMI categories. However, in the absence of validated perceived‐stress or anxiety scales, we cannot distinguish the relative contributions of metabolic status versus unmeasured psychosocial stressors to these chronic cortisol differences.

Hair progesterone levels in the first trimester were significantly lower in overweight participants compared to normal weight participants (*P* = 0.043, data not shown). Other hormones (hair testosterone and DHEA) did not differ between BMI categories.

Maternal serum cortisol levels during pregnancy (first, second, and third trimester) and at birth were not significantly different across GWG categories (Figure 4c). By 6 months postpartum, maternal cortisol levels were significantly higher in the excessive GWG group than in women with recommended GWG (*P* = 0.045, Figure 4c), with this difference being most pronounced among mothers of male infants.

Maternal hair cortisol, cortisone, as well as hormone ratios (cortisol/cortisone), and other markers such as hair testosterone, DHEA, and progesterone did not differ significantly between GWG groups (Table 1).

### 3.4 Placental data (Tables 3 and 4)

While overall placental weight was similar across groups, a notable difference emerged in the placenta's short‐distance circumference (Table 3). Participants categorized as overweight who were carrying a male fetus, had a larger circumference compared with normal weight (*P* = 0.013) participants. This subtle morphological change may reflect adaptations in placentas supporting male fetuses with rising maternal BMI.

There were no significant differences between BMI groups in placental HSD11B2 mRNA or the percentage of CpG methylation of the HSD11B2 promoter (Table 1), regardless of topographic placental localization. Peripheral and the combined central and peripheral HSD11B2 protein levels in placentas were significantly lower in obese participants compared to normal weight and overweight participants (Table 1). This mainly affected placenta from participants with female fetuses.

Although gross placental measures with respect to GWG were similar (Table 4), in addition, specific DNA methylation profiles in one of four investigated CpG sites (CpG4) revealed increased methylation of the HSD11B2 promoter (Figure 2), both peripherally and centrally in placental tissue from participants that gained excess weight compared to those below the recommended amount (Figure 2).

### 3.5 Associations between maternal anthropometric and maternal cortisol variables and birth outcomes (Table S2)

Maternal height was robustly and positively correlated with neonatal size; maternal height correlated with birth weight, head circumference, and birth length (Table S2A, *P* < 0.001). Maternal weight showed a modest positive association with birth weight (*r* = 0.187, *P* < 0.05), and total GWG was positively correlated with birth weight, head circumference, and birth length (*P* < 0.05). In contrast, net weight gain (i.e., gain adjusted for other factors like placental weight) and pre‐pregnancy BMI did not display significant associations with neonatal anthropometrics.

Placental weight was positively associated with birth weight, head circumference, and birth length (Table S2A, *P* < 0.001). Third trimester maternal cortisol levels were positively associated with birth length (*P* < 0.05), while cortisol measured at birth was strongly linked to cord‐blood cortisol (*P* < 0.001). When looking at combined peripheral CpG sites 1–4, methylation of the HSD11B2 promoter was positively correlated with birth length and negatively with the ponderal index (*P* < 0.05). Similarly, methylation at central CpG site 1 showed a significant positive correlation with head circumference (*P* < 0.05).

Sex‐specific analyses (indicated by colored annotations in Table S2A) show that some of these associations varied by newborn sex. For instance, maternal height and pre‐pregnancy weight were more strongly associated with birth weight and length in males, whereas for females, cortisol and epigenetic markers (e.g., central CpG methylation) show more pronounced associations.

#### 3.5.1 Maternal–infant associations at 6 months postpartum (Table S2)

Maternal height was positively associated with infant height at 6 months (Table S2B, *P* < 0.001) although correlations with infant weight and head circumference did not reach significance. Total GWG was modest but significantly associated with infant weight and infant BMI (*P* < 0.05). Among maternal BMI outcome measures, only total BMI gain correlated significantly with infant BMI (*P* < 0.05).

Notably, maternal first trimester hair cortisol levels were positively associated with several infant outcomes: weight, length, and weight gain (*P* < 0.05). Other stress measures did not show significant associations with postnatal outcomes.

Placental weight showed significant positive associations with infant weight (*P* < 0.05), head circumference (*P* < 0.001), and height (*P* < 0.05). HSD11B2 protein levels showed significant negative associations with infant weight and BMI (*P* < 0.05). Among the HSD11B2 methylation markers, methylation of peripheral CpG site 2 was significantly associated with infant head circumference (*P* < 0.05) and central CpG site 1 methylation was positively associated with infant height (*P* < 0.05).

#### 3.5.2 Neonatal–infant associations at 6 months postpartum (Table S2)

Birth weight demonstrated robust positive correlations with infant weight, head circumference, and length (Table S2C, *P* < 0.001) at 6 months. Similarly, neonatal birth length and head circumference were significantly associated with infant outcomes; head circumference at birth correlated with infant head circumference and infant height (*P* < 0.001). Neither the neonatal ponderal index nor fetal venous cortisol showed significant correlations with any of the 6‐month infant parameters.

### 3.6 Crude and adjusted linear regression model outcomes

#### 3.6.1 Neonatal outcomes and maternal anthropometrics (Table 5a)

**TABLE 5a:** Crude and adjusted linear regression analyses for maternal anthropometric variables versus neonatal anthropometric variables.

Outcome variable	Independent maternal variable	Crude			Adjusted^a^					
					Model I			Model II		
		B	(95% CI)	*P* value	B	(95% CI)	*P* value	B	(95% CI)	*P* value
Placental weight	Height	1.904	−0.859; 4.668	ns	0.021	−3.053; 3.095	ns	−0.821	−3.791; 2.150	ns
	Pre‐pregnancy weight^1^	570	−3446; 4586	ns	1668	−2392; 5729	ns	2709	−1196; 6614	ns
	Pre‐pregnancy BMI^1^	3855	−4760; 12 471	ns	3708	−4907; 12 322	ns	5279	−2999; 13 557	ns
	Weight at birth	0.213	−0.1088; 1.514	ns	−0.250	−1.590; 1.090	ns	−0.628	−1.919; 0.663	ns
	BMI at birth^1^	673	−2352; 3699	ns	813	−2252; 3874	ns	1417	−1524; 4358	ns
	Total weight gain	2.262	−1.030; 5.554	ns	1.203	−2.141; 4.547	ns	1.034	−2.159; 4.244	ns
	Total BMI gain	5.268	−4.012; 14.548	ns	3.348	−5.968; 12.664	ns	3.287	−5.621; 12.195	ns
	Net GWG	1.172	2.177; 4.520	ns	0.286	−3.069; 3.640	ns	0.374	−2.836; 3.584	ns
Birth weight^1^	Height	**16 365**	**8713; 24 018**	**<0.001**	**7240**	**1089; 13 391**	**0.021**	4481	−580; 9542	ns
	Pre‐pregnancy weight^1^	570	−3446; 4586	ns	1668	−2392; 5729	ns	2709	−1196; 6614	ns
	Pre‐pregnancy BMI^1^	3855	−4761; 12 471	ns	3708	−4907; 12 322	ns	5279	−2999; 13 557	ns
	Weight at birth	0.226	−1.061; 1.514	ns	−0.308	−1.628; 1.011	ns	−0.672	−1.945;0.601	ns
	BMI at birth^1^	−258 776	−2 116 102; 1 598 550	ns	−481 212	−2 343 641; 1 381 217	ns	−799 941	−2 590 349; 990 467	ns
	Total weight gain	2.597	−0.629; 5.823	ns	1.325	−1.987; 4.636	ns	1.178	−1.997; 4.352	ns
	Total BMI gain	6.447	−2.622; 15.515	ns	3.892	−5.296; 13.080	ns	3.890	−4.906;12.685	ns
	Net GWG	1.604	−1.694; 4.901	ns	0.556	−2.762; 3.875	ns	0.639	−2.542; 3.820	ns
Head circumference^1^	Height	−0.160	−0.384; 0.064	ns	−0.202	−0.452; 0.047	ns	−0.231	−0.481; 0.020	ns
	Pre‐pregnancy weight^1^	−124.6	−457.4; 208.2	ns	−99.9	−447.7; 247.8	ns	−72.4	−422.6; 277.7	ns
	Pre‐pregnancy BMI^1^	−644.5	−1354.8; 65.9	ns	−610.8	−1346.1; 124.5	ns	−579.7	−1317.9; 158.5	ns
	Weight at birth	0.59	0.258; −0.044	ns	0.051	−0.057; 0.159	ns	0.041	−0.067; 0.150	ns
	BMI at birth^1^	**156 061**	**6959; 305 162**	**0.040**	145 324	−9077; 299 725	ns	136 647	−18 429; 291 723	ns
	Total weight gain	0.127	−0.131; 0.386	ns	0.125	−0.147; 0.396	ns	0.103	−0.169; 0.375	ns
	Total BMI gain	0.435	−0.301; 1.171	ns	0.436	−0.331; 1.203	ns	0.389	−0.378; 1.156	ns
	Net GWG	0.023	−0.019; 0.065	ns	0.000	−0.034; 0.035	ns	0.000	−0.032; 0.032	ns
Length^1^	Height	**0.016**	**0.008; 0.023**	**<0.001**	**0.007**	**0.000; 0.014**	**0.039**	0.006	−0.001; 0.012	ns
	Pre‐pregnancy weight^1^	**−12.275**	**−24.047; −0.503**	**0.041**	−5.490	−14.829; 3.849	ns	−3.194	−12.304; 5.915	ns
	Pre‐pregnancy BMI^1^	−5.442	−31.075; 20.191	ns	−3.386	−23.350; 16.579	ns	0.480	−18.890; 19.849	ns
	Weight at birth	**0.005**	**0.001; 0.009**	**0.006**	0.002	−0.001; 0.004	ns	0.001	−0.002; 0.004	ns
	BMI at birth^1^	3078	−2234; 8990	ns	385.4	−3788.6; 4559.4	ns	−474.8	−4524.3; 3574.6	ns
	Total weight gain	0.009	0.000; 0.018	ns	0.000	−0.007; 0.007	ns	−0.001	−0.008; 0.006	ns
	Total BMI gain	0.016	−0.010; 0.042	ns	−0.004	−0.024; 0.016	ns	−0.006	−0.026, 0.014	ns
	Net GWG	0.005	−0.005; 0.016	ns	−0.001	−0.009; 0.007	ns	−0.001	−0.09; 0.006	ns
Ponderal index^1^	Height	7.587E‐5	−0.001; 0.001	ns	0.000	−0.001; 0.001	ns	0.000	−0.001; 0.001	ns
	Pre‐pregnancy weight^1^	0.78	−1.108; 1.264	ns	−0.239	−1.442; 0.963	ns	−0.405	−1.610; 0.801	ns
	Pre‐pregnancy BMI^1^	0.457	−2.094; 3.008	ns	−0.048	−2.611; 2.514	ns	−0.353	−2.915; 2.209	ns
	Weight at birth	−7.327E‐5	0.000; 0.000	ns	2.763E‐5	0.000; 0.000	ns	7966E‐5	0.00; 0.000	ns
	BMI at birth^1^	−161.2	−695.6; 373.1	ns	−51.2	−589.2; 486.8	ns	6.498	−531.6; 544.6	ns
	Total weight gain	0.000	−0.001; 0.000	ns	0.000	−0.001; 0.001	ns	0.000	−0.005; 0.001	ns
	Total BMI gain	−0.001	−0.004; 0.001	ns	−0.001	−0.004; 0.001	ns	−0.001	−0.004; 0.001	ns
	Net GWG	0.000	−0.001; 0.001	ns	0.000	−0.001; 0.001	ns	0.000	−0.001; 0.001	ns

*Note*: BMI, calculated as weight in kilograms divided by the square of height in meters. Net GWG = genuine maternal weight gain in kg (total GWG‐birth weight‐placental weight). BMI, body mass index; CI, confidence interval; GWG, gestational weight gain; ns, non‐significant. ^a^Model I: Adjusted for maternal age, gestational age in completed weeks, parity, smoking during pregnancy, ethnic origin. Model II: Model I adjustment plus infant's sex and macrosomia >4000 g. ^1^For regression analyses, normal distribution was achieved for respective variables by transformation using the Box‐Cox transformation (Hemmerich).^50^ StatistikGuru: Box‐Cox Powertransformation berechnen. Retrieved from https://statistikguru.de/rechner/box‐cox.html.

Neonatal birth weight was positively associated with maternal height in the crude model (Table 5a, *P* < 0.001) and remained significant after adjustment for maternal age, gestational age, parity, smoking, and ethnic origin (Model I *P* = 0.021). However, when further adjusting for infant sex and macrosomia (Model II), the association was no longer significant (Table 5a).

None of the maternal predictors, including height, pre‐pregnancy weight, or BMI were associated with neonatal head circumference in adjusted models. A significant association was observed in the crude analysis for BMI at birth (*P* = 0.040) but this did not persist once adjustments were made (Model I and II).

Maternal height showed a positive relationship with birth length. In the crude analysis, height was positively associated with length (*P* < 0.001) but was not significant in Model I or II.

No significant relationships were observed between maternal anthropometrics (height, weight, BMI, or weight gain measures) and neonatal ponderal index across any of the models.

Maternal outcomes were also grouped by BMI and GWG, where we compared maternal and infant data to a reference group consisting of appropriate‐for‐gestational‐age and non‐macrosomic infants. Percentages were calculated relative to the reference group, which is defined as infants appropriate for gestational age and without macrosomia (Figure 5). Participants in the overweight pre‐pregnancy BMI category were at a higher risk of having infant macrosomia and overweight.

**FIGURE 5:**
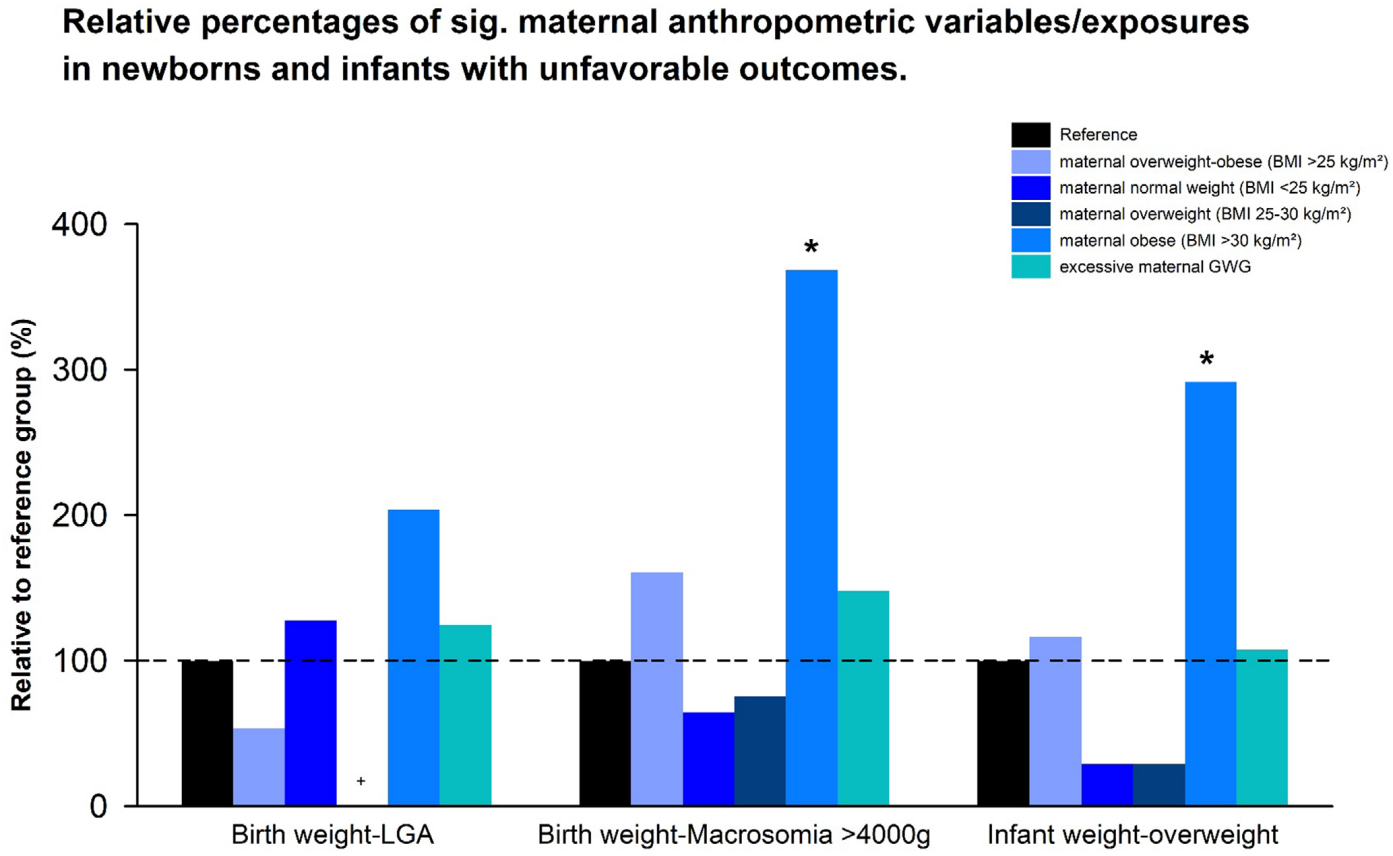
Proportion of participants that have newborns with unfavorable outcomes relative to respective reference groups. Maternal overweight, obesity and excessive GWG are associated with significantly increased risks of adverse infant outcomes, particularly macrosomia and early life overweight. The strongest associations were observed in the overweight (BMI 25–30) groups. Reference group consists of appropriate‐for‐gestational age and non‐macrosomic infants. Significant differences are indicated as *P < 0.05. BMI, body mass index; GWG, gestational weight gain; LGA, large for gestational age. ^+^ *n* = 0.

#### 3.6.2 Infant anthropometric outcomes at 6 months and maternal anthropometrics (Table 5b)

**TABLE 5b:** Crude and adjusted linear regression analyses for maternal anthropometric variables versus child anthropometric outcome variables at 6 months postpartum.

Outcome variable 6 months pp	Independent maternal variable	Crude			Adjusted^a^					
					Model I			Model II		
		B	(95% CI)	*P* value	B	(95% CI)	*P* value	B	(95% CI)	*P* value
Weight	Height	27.56	−7.035; 62.16	ns	28.03	−6.334; 62.40	ns	16.36	−19.93; 47.66	ns
	Pre‐pregnancy weight^1^	−41 617	−89 531; 6298	ns	**−47 177**	**−93 849; −505.8**	**0.048**	−34 315	−77 288; 8657	ns
	Pre‐pregnancy BMI^1^	−57 307	−155 411; 40 798	ns	−72 559	−169 426; 24 308	ns	−55 048	−143 363; 33 267	ns
	Weight at birth	**16.58**	**3.839; 29.32**	**0.011**	**16.84**	**4.419; 29.27**	**0.009**	**13.43**	**2.082; 24.78**	**0.021**
	BMI at birth^1^	−24 848	−55 694; 5997	ns	−25 950	−56 391; 4991	ns	−21 101	−48 666; 6463	ns
	Total weight gain	31.48	−3.322; 66.28	ns	27.55	−8.169; 63.28	ns	22.09	−9.895; 54.07	ns
	Total BMI gain	83.38	−19.50; 186.2	ns	71.08	−34.04; 176.2	ns	60.78	−33.014; 154.6	ns
	Net GWG	17.84	−26.54; 62.22	ns	9.779	−36.37; 55.93	ns	15.85	−25.74; 57.44	ns
Height^1^	Height	**2.633**	**1.600; 3.666**	**<0.001**	**1.895**	**0.856; 2.934**	**<0.001**	**1.872**	**0.822; 2.923**	**<0.001**
	Pre‐pregnancy weight^1^	**−2951**	**−4526; −1375**	**<0.001**	**−2555**	**−3994; −1115**	**<0.001**	**−2497**	**−3957; −1037**	**<0.001**
	Pre‐pregnancy BMI^1^	−3025	−6519; 470.4	ns	−3426	−6561; −290.6	ns	**−3262**	**−6430;‐94.7**	**0.044**
	Weight at birth	**0.962**	**0.480; 1.444**	**<0.001**	**0.701**	**0.252; 1.151**	**0.002**	**0.684**	**0.227; 1.141**	**0.004**
	BMI at birth^1^	641 126	−92 617; 13 744 869	ns	503 311	−161 270; 1 167 892	ns	470 367	−200 631; 1 141 365	ns
	Total weight gain	0.644	−0.623; 1.912	ns	−0.293	−1.459; 0.873	ns	−0.291	−1.465; 0.882	ns
	Total BMI gain	0.175	−3.448; 3.798	ns	−2.047	−5.333; 1.238	ns	−2.024	−5.328; 1.279	ns
	Net GWG	0.008	−1.384; 1.400	ns	−0.663	−1.940; 0.614	ns	−0.631	−1.912; 0.650	ns
Head circumference	Height	0.42	−0.007; 0.090	ns	0.040	−0.010; 0.091	ns	0.023	−0.022; 0.068	ns
	Pre‐pregnancy weight^1^	−37.74	−106.4; 30.92	ns	−39.39	−110.3; 31.50	ns	−19.39	−82.76; 43.98	ns
	Pre‐pregnancy BMI^1^	−39.40	−181.4; 102.6	ns	−45.19	−193.7; 103.3	ns	−16.96	−148.5; 114.6	ns
	Weight at birth	0.010	−0.009; 0.029	ns	0.011	−0.008; 0.030	ns	0.005	−0.012; 0.022	ns
	BMI at birth^1^	−6.638	−51.70; 38.41	ns	−10.16	−56.94; 36.63	ns	−1.13	−42.24; 39.97	ns
	Total weight gain	0.005	−0.046; 0.056	ns	0.010	−0.045; 0.066	ns	−0.003	−0.051; 0.045	ns
	Total BMI gain	−0.003	−0.154; 0.148	ns	0.012	−0.150; 0.174	ns	−0.019	−0.160; 0.122	ns
	Net GWG	−0.032	−0.095; 0.032	ns	−0.031	−0.101; 0.038	ns	0.037	−0.041; 0.115	ns
BMI^1^	Height	**2.565**	**1.558; 3.571**	**<0.001**	**1.845**	**0.833; 2.858**	**<0.001**	**1.823**	**0.800; 2.847**	**<0.001**
	Pre‐pregnancy weight^1^	**−2784**	**−4409; −1339**	**<0.001**	**−2488**	**−3891; −1086**	**<0.001**	**−2433**	**−3855; −1010**	**<0.001**
	Pre‐pregnancy BMI^1^	−2946	−6350; 459	ns	**−3337**	**−6392; −282.8**	**0.032**	**−3178**	**−6264; −92.3**	**<0.001**
	Weight at birth	**0.937**	**−0.467; 1.407**	**<0.001**	**0.683**	**0.245; 1.121**	**0.002**	**0.666**	**0.221; 1.111**	**0.004**
	BMI at birth^1^	624 412	−90 381; 1 339 205	ns	490 201	−157 229; 1 137 630	ns	458 198	−195 489; 1 111 886	ns
	Total weight gain	0.627	−0.607; 1.862	ns	−0.286	−1.421; 0.850	ns	−0.284	−1.427; 0.859	ns
	Total BMI gain	0.170	−3.360; 3.699	ns	−1.995	−5.196; 1.206	ns	−1.973	−5.191; 1.246	ns
	Net GWG	0.008	−1.348; 1.364	ns	−0.646	−1.890; 0.598	ns	−0.615	−1.863; 0.633	ns
Weight gain	Height	11.70	−18.51; 41.91	ns	12.36	−17.83; 42.56	ns	5.893	−23.6; 34.84	ns
	Pre‐pregnancy weight^1^	−34 720	−76 122; 6681	ns	−39 463	−80 018; 1092	ns	−38 345	−77 520; 829.2	ns
	Pre‐pregnancy BMI^1^	−59 630	−143 971; 24 710	ns	−72 377	−155 987; 11 232	ns	−75 712	−155 828; 4405	ns
	Weight at birth	**13.70**	**2.664; 24.73**	**0.016**	**14.48**	**3.698; 25.27**	**0.009**	**13.71**	**3.369; 24.05**	**0.010**
	BMI at birth^1^	−24 293	−50 792; 2206	ns	**−26 321**	**−52 535; −106.3**	**0.049**	**−27 063**	**−52 017; −2108**	**0.034**
	Total weight gain	26.27	−3.806; 56.34	ns	25.77	−5137; 56.67	ns	21.35	−8.006; 50.71	ns
	Total BMI gain	76.61	−11.98; 165.2	ns	73.56	−17.15; 164.3	ns	63.59	−22.34; 149.5	ns
	Net GWG	19.58	−18.14; 57.31	ns	16.48	−23.01; 55.97	ns	19.17	−18.39; 56.72	ns

*Note*: BMI, calculated as weight in kilograms divided by the square of height in meters. Net GWG = genuine maternal weight gain in kg (total GWG‐birth weight‐placental weight). BMI, body mass index; CI, confidence interval; GWG, gestational weight gain; ns, non‐significant; pp, postpartum. ^a^Model I: Adjusted for maternal age, gestational age in completed weeks, parity, smoking during pregnancy, ethnic origin. Model II: Model I adjustment plus infant's sex and macrosomia >4000 g. ^1^For regression analyses, normal distribution was achieved for respective variables by transformation using the Box‐Cox transformation (Hemmerich).^50^ StatistikGuru: Box‐Cox Powertransformation berechnen. Retrieved from https://statistikguru.de/rechner/box‐cox.html.

Birth weight emerged as a strong positive predictor of infant weight at 6 months. In the crude model, higher birth weight was associated with increased infant postnatal weight (*P* = 0.011), and this association persisted in Model I (*P* = 0.009) and Model II (*P* = 0.021). In contrast, a higher maternal pre‐pregnancy weight was associated with lower infant weight in Model I (*P* = 0.048).

Maternal height was a consistent positive predictor of infant height at 5 months (Model II *P* < 0.001). Higher pre‐pregnancy weight (Model II *P* < 0.001) and BMI (Model II *P* = 0.044) in contrast, were inversely associated with infant height.

Maternal height positively predicted infant BMI at 6 months (Model II *P* < 0.001), but both higher pre‐pregnancy weight (Model II *P* < 0.001) and BMI (Model II *P* < 0.001) showed significant inverse associations. Birth weight was also positively correlated with infant BMI (Model II *P* = 0.004). Birth weight positively predicted infant weight gain (Model II *P* = 0.010). Across all models, maternal anthropometric measures did not show significant associations with infant head circumference or postnatal weight gain at 6 months.

### 3.7 Maternal determinants of anthropometric birth and cord‐blood hormone outcomes (Table 6)

Maternal cortisol showed distinct associations with neonatal outcomes depending on timing, and acute versus chronic HPA markers (Table 6a). Higher maternal hair cortisol (a marker of chronic stress) in both the first and third trimesters was associated with increased birth weight, whereas elevated maternal serum cortisol in the third trimester (a marker of acute stress) was inversely associated with birth weight (*P* = 0.020; Adj. Sr^2^ = 0.091). These findings underscore the importance of differentiating acute and chronic measures and their timing when evaluating impacts on fetal growth.

**TABLE 6a:** Maternal categorial predictor variables affecting anthropometric birth outcomes, cord‐blood hormones and placental parameters.

Outcome variable	Predictor variable^a^	B	(95% CI)	*P* value	Adj. Sr^2^
Placental weight	Maternal cortisol second trimester ≥ 90th percentile	2.043	0.668; 3.419	0.006	0.319
	BMI pre‐pregnancy obese	114.369	20.025; 208.713	0.020	0.213
Birth weight	Cesarean delivery	−751.2	−1054.4; −447.9	<0.001	0.445
	Hair cortisol third ≥90th percentile	661.4	306.7; 1016.1	<0.001	0.252
	Hair cortisol first ≥90th percentile	483.3	152.0; 814.5	0.006	0.154
	Cortisol third ≥90th percentile	−4.328	−7.640; −1.017	0.013	0.124
Head circumference	Hair cortisol second ≥90th percentile	1.858	0.623; 3.093	0.005	0.282
	BMI prepregnancy overweight	−0.882	−1.737; −0.027	0.044	0.132
Relative birth weight	Maternal cortisol second ≥90th percentile	−0.045	−0.073; −0.016	0.004	0.375
Ponderal index	Maternal cortisol first trimester ≥90th percentile	−0.007	−0.012; −0.002	0.006	0.202
	Nulliparous	−0.290	−0.494; −0.085	0.008	0.183
	Hair cortisol first ≥90th percentile	0.341	0.057; 0.625	0.021	0.131
	Excessive weight gain	0.193	0.001; 0.386	0.049	0.092

*Note*: BMI, calculated as weight in kilograms divided by the square of height in meters. Forward stepwise regression analyses with key maternal general, anthropometric, and metabolic parameters in (clinically) relevant categories were then performed to identify major predictors for critical birth outcomes. Unstandardized B coefficients with 95% CI and adjusted semipartial correlation coefficients (Sr^2^), which estimate the contribution of each factor to the variance of the outcome variable, are shown. BMI, body mass index; CI, confidence interval; GWG, gestational weight gain. ^a^Included categorial variables: maternal age < 20 years (yes/no), maternal age 20–30 years (yes/no), maternal age 30–40 years (yes/no), maternal age > 40 years (yes/no), ethnicity European (non/yes), parity nulliparous (no/yes), smoking in pregnancy (no/yes), mode of delivery cesarean section (no/yes), gestational age preterm delivery <37^+0^ weeks (no/yes), infant's sex female (no/yes), maternal pre‐pregnancy BMI 20–25 (no/yes), pre‐pregnancy BMI 25–30 (no/yes), pre‐pregnancy BMI >30 (no/yes), inadequate GWG (no/yes), excessive GWG (no/yes), maternal cortisol first/s/third trimester ≥90th percentile, maternal cortisol at delivery ≥90th percentile, maternal hair cortisol first/s/third trimester ≥90th percentile, HSD11B2 protein level peripheral/central/peripheral+central ≥90th percentile.

Elevated maternal second trimester serum cortisol levels were associated with increased placental weight (*P* = 0.006, Adj. Sr^2^ = 0.319) and a reduced birth weight to placental weight ratio (*P* = 0.004, Adj. Sr^2^ = 0.375). Cesarean delivery was associated with lower birth weight (*P* < 0.001; Adj. Sr^2^ = 0.318), while nulliparity correlated with a reduced ponderal index (*P* = 0.008; Adj. *r* ^2^ = 0.183), indicating that both obstetric procedures and maternal reproductive history may contribute to neonatal anthropometry.

At 6 months, female sex was a dominant influencing factor on infant weight (Table 6b, *P* < 0.001; Adj. Sr^2^ = 0.379), head circumference (*P* = 0.002; Adj. Sr^2^ = 0.309), BMI (*P* = 0.003; Adj. Sr^2^ = 0.336), and overall weight gain (*P* = 0.001; Adj. Sr^2^ = 0.379). Additionally, maternal factors, such as pre‐pregnancy obesity (*P* = 0.037; Adj. Sr^2^ = 0.125) and second‐trimester hair cortisol (*P* = 0.008; Adj. Sr^2^ = 0.279), continued to play a significant role in shaping infant growth.

**TABLE 6b:** Maternal categorial predictor variables affecting anthropometric child outcomes at 6 months postpartum.

Outcome variable 6 months pp	Predictor variable^a^	B	(95% CI)	*P* value	Adj. Sr^2^
Weight	Female sex	1324.8	617.4; 2032.3	<0.001	0.379
	BMI pre‐pregnancy obese	1004.0	68.1; 1939.9	0.037	0.125
Height	Hair cortisol second ≥90th percentile	5.476	1.582; 9.370	0.008	0.279
Head circumference	Female sex	1.945	1.052; 2.838	<0.001	0.403
	HSD11B2 protein peripheral ≥90th percentile	−0.016	−0.028; −0.005	0.006	0.184
	BMI pre‐pregnancy obese	1.218	0.004; 2.432	0.049	0.085
BMI	Female sex	2.062	0.718; 3.342	0.003	0.336
Weight gain	Female sex	1192	517.5; 1866.9	0.001	0.379

*Note*: BMI, calculated as weight in kilograms divided by the square of height in meters. Forward stepwise regression analyses with key maternal general, anthropometric, and metabolic parameters in (clinically) relevant categories were then performed to identify major predictors for critical birth outcomes. Unstandardized B coefficients with 95% CI and adjusted semipartial correlation coefficients (Sr^2^), which estimate the contribution of each factor to the variance of the outcome variable, are shown. Net GWG = genuine maternal weight gain in kg (total GWG‐birth weight‐placenta weight), relative birth weight (birth weight to height ratio). BMI, body mass index; CI, confidence interval; GWG, gestational weight gain; pp, postpartum. ^a^Included categorial variables: maternal age < 20 years (yes/no), maternal age 20–30 years (yes/no), maternal age 30–40 years (yes/no), maternal age > 40 years (yes/no), ethnicity European (non/yes), parity nulliparous (no/yes), smoking in pregnancy (no/yes), mode of delivery cesarean section (no/yes), gestational age preterm delivery <37^+0^ weeks (no/yes), infant's sex female (no/yes), maternal pre‐pregnancy BMI 20–25 (no/yes), pre‐pregnancy BMI 25–30 (no/yes), pre‐pregnancy BMI > 30 (no/yes), inadequate GWG (no/yes), excessive GWG (no/yes), maternal cortisol first/s/third trimester ≥90th percentile, maternal cortisol at delivery ≥90th percentile, maternal hair cortisol first/s/third trimester ≥90th percentile, HSD11B2 protein level peripheral/central/peripheral+central ≥90th percentile.

## 4 DISCUSSION

It is now well recognized that early life factors influence the risk for noncommunicable diseases (NCDs) later in life.^1^, ^2^ Parental exposures—particularly maternal nutrition, adiposity, and stress—affect fetal programming and contribute to intergenerational transmission of cardiometabolic risk.^10^ Higher maternal BMI, excessive GWG, and adiposity are consistently linked with childhood obesity and metabolic syndrome.^5^, ^6^, ^7^, ^8^, ^9^ Rising rates of overweight and obesity in women of reproductive age therefore raise concern for adverse pregnancy outcomes and long‐term child health.^11^, ^12^, ^13^, ^14^, ^15^

### 4.1 Maternal anthropometrics and gestational weight gain: Implications for neonatal growth and postpartum outcomes

In our study, participants largely maintained their pre‐pregnancy weight category through pregnancy and postpartum. While total GWG did not differ greatly between BMI groups, net GWG (adjusted for fetal/placental weight) was lower in overweight and obese participants than in normal‐weight participants. Excessive GWG was common in obese participants but also observed in normal‐weight participants. Six months postpartum, weight retention was highest among obese mothers.

Traditional maternal measures showed limited associations with neonatal outcomes. After adjustment for confounders, maternal height emerged as the strongest predictor of birth length and later infant size, whereas pre‐pregnancy BMI and weight showed inconsistent associations. Birth weight remained a robust predictor of early growth, supporting calls for pre‐ and postconception counseling across the BMI spectrum.

### 4.2 Neonatal anthropometry and early infant outcomes

Neonatal growth measures such as birth weight, length, and head circumference are reliable indicators of the intrauterine environment.^5^, ^9^ In our dataset, although the mean values of these neonatal anthropometrics did not differ significantly across maternal BMI groups, the distribution revealed important deviations. For instance, the rate of macrosomia was markedly higher among obese participants compared with overweight participants. In male infants, those born to participants with below‐recommended GWG displayed significantly lower birth weights compared with those from recommended or excessive GWG groups. Despite these group differences in birth weight categories, the mean infant anthropometric values at 6 months postpartum did not show significant differences, hinting at possible postnatal compensatory growth mechanisms.^42^, ^52^

Maternal height emerged as the most consistent positive predictor of neonatal growth parameters, particularly birth length and weight, confirming prior observations that taller mothers tend to have larger neonates, likely due to shared genetic and nutritional influences.^53^ The attenuation of this association after adjusting for infant sex and macrosomia suggests that fetal size determinants interact with maternal stature, but are also modulated by fetal growth programming and sex‐specific patterns.^54^

In contrast, pre‐pregnancy BMI and weight showed no consistent or significant associations with birth weight, head circumference, or ponderal index, a pattern divergent from large cohort studies (e.g., Hinkle et al.^55^), which have reported linear associations between maternal obesity and macrosomia. This discrepancy could be due to population‐specific effects, sample size, or that maternal gestational diabetes mellitus (GDM) was explicitly excluded from our study and could mediate the role of hormonal and placental adaptations in other cohorts.

By 6 months postpartum, maternal anthropometry exerted differential influences on infant growth. Notably, pre‐pregnancy weight and BMI were inversely associated with infant height and BMI, even after controlling for confounders. This inverse association runs counter to the hypothesis that maternal obesity confers sustained increased growth risk in offspring,^5^ and may suggest a delayed or compensatory growth response or the influence of postnatal environmental and metabolic factors (e.g., breastfeeding, maternal–infant interaction, inflammatory profiles).

Importantly, birth weight strongly predicted infant weight, height and BMI at 6 months, in line with the concept of fetal programming and the persistence of early growth patterns. The consistently positive influence of maternal height on infant height and BMI highlights the interplay between genetic determinants and intrauterine nutritional adequacy.^56^ Interestingly, no maternal anthropometric variables predicted infant head circumference or postnatal weight gain, suggesting that postnatal brain growth and early weight dynamics may be buffered against maternal size or regulated by distinct mechanisms, such as neuroendocrine and metabolic signals.^57^, ^58^

### 4.3 The materno‐fetal stress axis: Endocrine and epigenetic considerations

A central focus of our investigation was the potential role of hypothalamic–pituitary–adrenal (HPA) axis activity and cortisol in mediating the effects of maternal adiposity and GWG. In healthy pregnancies, overall cortisol secretion rises gradually, with a characteristic morning peak,^59^, ^60^ and stress reactivity tends to decline as gestation progresses, possibly acting as a protective adaptation against excessive fetal cortisol exposure.^61^, ^62^ However, in obese pregnancies, this regulatory mechanism may be altered. Although our data showed that absolute maternal serum cortisol levels during pregnancy did not differ significantly between BMI groups, the dynamic increase from the second to the third trimester was significantly more pronounced in obese participants. Despite increased hair cortisol and cortisone levels in overweight and obese women, indicating increased chronic stress, our blood‐based cortisol measurements did not confirm a state of systemic hypercortisolemia, a discrepancy that underscores the importance of the biological markers used for HPA axis assessment.^63^, ^64^, ^65^

Moreover, the anticipated rise in fetal (cord blood) cortisol in response to maternal hypercortisolemia was not evident, which might be due to the placental barrier function mediated by HSD11B2,^66^, ^67^ since we did see subtle differences in placental HSD11B2 methylation, particularly in female infants. We showed that increased methylation of HSD11B2 at combined CpG sites 1–4 correlated with birth length and suggests a key epigenetic mechanism through which maternal stress may influence fetal development. Hypermethylation of HSD11B2 promoter regions might generally lead to decreased enzyme expression,^68^ reducing enzymatic inactivation of cortisol to cortisone and, therefore, increasing fetal exposure.^69^ However, in this cohort, greater methylation correlated with enhanced fetal growth, potentially indicating context‐specific methylation patterns (e.g., affecting activating or inactivating transcription factor binding sites) or compensatory mechanisms. Experimental studies have demonstrated that adverse maternal conditions, such as nutritional deficits and elevated cortisol, can induce epigenetic modifications in the placenta, thereby altering its nutrient transport capacity and potentially programming long‐term disease risk in the offspring.^70^, ^71^, ^72^ These may still reflect important sex‐specific adaptations that warrant further investigation.

One of the striking findings is the significantly higher maternal cortisol levels at 6 months postpartum in participants with excessive GWG compared to those with recommended GWG. Elevated cortisol in the postpartum period could be a marker of prolonged HPA axis activation. The fact that altered cortisol dynamics extended into the postpartum period, with evidence of persistent weight retention suggests that the impacts of maternal HPA axis dysregulation do not resolve immediately after birth. These ramifications underscore the need for clinicians to monitor not only peripartum weight gain but also the postpartum hormonal milieu as part of routine follow‐up, potentially opening communication for early interventions in mood and metabolic support.

### 4.4 The role of the placenta in modulating fetal exposure

The placenta is central to ensuring fetal homeostasis by regulating the exchange of nutrients and hormones, including cortisol.^73^, ^74^ In this study, maternal pre‐pregnancy BMI, weight, and height did not significantly correlate with placental weight in either crude or adjusted models. This finding aligns with some literature suggesting that placental growth is not linearly associated with maternal adiposity but may instead reflect more complex interactions, including placental efficiency, vascular development, and inflammatory signaling.^73^, ^75^ In our study, gross placental morphology (e.g., weight and circumferences) was largely similar across maternal BMI and GWG groups, with the exception of a larger short‐distance circumference in overweight participants.

### 4.5 Mechanistic and clinical considerations

Maternal obesity is known to impair leptin signaling, in part by inducing leptin resistance, which disrupts inhibitory feedback on the HPA axis, resulting in chronically elevated cortisol levels.^76^, ^77^ Such endocrine perturbations not only contribute to excessive GWG but may also alter nutrient partitioning and fetal metabolism. Although we did not consistently observe hypercortisolemia, elevated hair cortisol levels in obese participants suggests a state of chronic elevated glucocorticoid exposure with potential implications for fetal growth and development.^63^, ^64^ Subtle epigenetic changes in HSD11B2 promoter methylation might be involved, mechanistically.^66^, ^78^

Sex‐specific differences appeared evident, with male infants showing consistently higher weight, BMI, and head circumference at 6 months, suggesting early sexual dimorphism in growth patterns, possibly mediated by placental function.^54^ The role of fetal sex in modulating the impact of maternal cortisol and adiposity further emphasizes the importance of sex‐stratified analyses in perinatal research. Clinically, these findings reinforce the need for comprehensive parental preconception and prenatal care strategies that include nutritional and stress management interventions across all BMI categories.^34^ Incorporating chronic stress biomarkers such as hair cortisol into routine assessments may provide a broader perspective on maternal HPA axis status than conventional serum measurements.^79^, ^80^

### 4.6 Study limitations

We recognize this study had limitations. The overall sample size, while moderate for the primary analyses, was relatively small, especially for detailed sex‐specific subgroup analyses on hormonal and epigenetic outcomes. We therefore present our sex‐stratified results with caution, recognizing that small subgroup sizes reduce statistical power. Our single‐center design at Charité University Berlin may limit external validity; single‐center studies frequently lack the broader external validity required to support generalizable conclusions. Likewise, our strict exclusion criteria (e.g., excluding participants with gestational diabetes, severe comorbidities or other complications) were intended to isolate the distinct effects of maternal overweight but further constrain the study population. Such extensive exclusion criteria have been shown to restrict the diversity of participants and thereby limit the applicability of findings to the general population. In other words, our results may not fully generalize to participants with higher‐risk pregnancies who were excluded by design. Our study also has inherent measurement constraints; pre‐pregnancy weight was based on retrospective self‐report, which can underestimate true weight. For example, participants often report pre‐pregnancy weight about 1–2 kg lower than their measured weight^81^ potentially leading to misclassification of BMI. Similarly, we measured cortisol in different biological matrices (maternal hair vs. blood), which capture stress levels over different time frames. Hair cortisol reflects chronic exposure over months, whereas serum cortisol is a point‐in‐time measure; these are not directly interchangeable. In fact, one study found no significant correlation between hair cortisol concentrations and single‐point serum or saliva cortisol levels.^82^ Therefore, discrepancies between hair and blood cortisol in our data may partly reflect these methodological differences. Finally, our epigenetic findings (placental HSD11B2 promoter methylation) should be viewed as preliminary. We measured methylation at selected single CpG sites in the HSD11B2 promoter, but methylation can vary across different genomic regions and placental locations. Importantly, DNA methylation changes do not always imply functional changes in gene activity. For instance, a prior study observed only a moderate inverse correlation between HSD11B2 promoter methylation and its mRNA expression in placenta.^83^ Despite using multivariable regression to adjust for known confounders, residual confounding may persist. As noted in observational study methodology, unmeasured or imperfectly measured variables can bias associations.^84^ For example, an important limitation is the lack of standardized psychological assessments (e.g., perceived stress scales, anxiety or depression screenings, and detailed socioeconomic indices). Although we excluded women with diagnosed depression and major comorbidities and adjusted for several demographic and obstetric variables, residual confounding by psychosocial stress and socioeconomic context cannot be ruled out, for example, women with a high BMI may experience more stress in pregnancy partly because they are repeatedly told that their weight makes the pregnancy “high risk” or “bad,” which can feel stigmatizing and blaming.^85^ In summary, these limitations—including limited subgroup power, single‐center sampling, selection biases, measurement constraints, and possible residual confounding—underscore that our findings should be interpreted with appropriate caution.

## 5 CONCLUSIONS AND FUTURE DIRECTIONS

In summary, our study reinforces the critical role of maternal BMI and GWG as determinants of neonatal growth and early childhood outcomes while demonstrating the complex involvement of the materno‐fetal stress axis. The current data suggests a more nuanced, non‐linear relationship between maternal body composition and offspring anthropometry, both at birth and at 6 months postpartum. Our data suggest that pre‐pregnancy BMI exerts the more pervasive influence on antenatal HPA‐axis dysregulation and associated neonatal growth deviations, while excessive GWG may amplify or prolong these effects, particularly in the postpartum period and via epigenetic fine‐tuning of placental glucocorticoid metabolism. These findings reinforce the emerging view that maternal body size, in isolation, may not consistently predict offspring growth without considering hormonal milieu, stress markers, placental function, and broader metabolic adaptations. Future studies should integrate validated psychometric instruments and detailed socioeconomic measures alongside biological markers of HPA‐axis function and placental epigenetics to disentangle more precisely the respective contributions of maternal adiposity and psychosocial stress to stress‐axis dysregulation in pregnancy.

### 5.1 Key conclusions

1. Maternal anthropometrics: Overweight and obese participants consistently demonstrate excess weight and are prone to excessive GWG. Even though total GWG may be similar across groups, the net gain and postpartum weight retention are notably higher in obese mothers.

2. Neonatal and postnatal growth: Although group mean values of neonatal anthropometrics do not differ markedly with maternal BMI, the incidence of outcomes such as macrosomia and SGA varies, emphasizing that altered fetal growth regulation is present in specific subgroups.

3. HPA axis dynamics and placental function: Elevated hair cortisol and altered placental HSD11B2 epigenetics imply that chronic maternal stress may influence fetal development, even if blood‐based cortisol levels remain within normal ranges.

4. Clinical implications: These data underscore the importance of antenatal counseling and interventions that focus on both nutritional and stress management for all expectant mothers, not only those classified as obese. Future work integrating multi‐omic approaches and longer‐term outcome studies will further clarify the mechanistic pathways linking these early exposures to later disease risk. Future studies could further elucidate whether interventions that modulate stress responses or improve placental function (such as nutritional counseling, stress management techniques, or targeted pharmacological interventions) might mitigate these risks. Understanding these hormonal dynamics in greater detail may lead to more individualized care plans that address both the immediate postpartum period and the longer‐term health of both mother and child.

## AUTHOR CONTRIBUTIONS

Thorsten Braun: Conceptualization, data curation, formal analysis, investigation, methodology, project administration, resources, visualization, writing–original draft. Marie Hofmann: Investigation, methodology, review and editing. Rebecca Rancourt: Investigation, methodology, validation, review and editing. Julia Sommer: Data curation, formal analysis, investigation, validation, review and editing. Alexandra Kühn: Investigation, validation, review and editing. Loreen Ehrlich: Investigation, validation, review and editing. Thomas Ziska: Investigation, validation, review and editing. Kerstin Melchior: Investigation, validation, review and editing. Karen Schellong: Investigation, validation, review and editing. Wolfgang Henrich: Validation, review and editing. Sonja Entringer: Validation, review and editing. Katherine Margaret Kennedy: Validation, review and editing. Andreas Plagemann: Conceptualization, investigation, methodology, project administration, resources, visualization, review and editing. Deborah Magdalena Sloboda: Conceptualization, investigation, methodology, project administration, resources, visualization, review and editing.

## FUNDING INFORMATION

T.B. is supported by Deutsche Forschungsgemeinschaft grant no. BR2925/10‐1. A.P. is supported by Deutsche Forschungsgemeinschaft grant no. PL241/16‐1. D.M.S. is supported by the Canada Research Chairs Program.

## CONFLICT OF INTEREST STATEMENT

No potential conflict of interest was reported by the author(s).

## Supporting information


**Data S1:** Supporting Information.

**Data S2:** Supporting Information.

## Data Availability

Data available on request from the authors.
