# Supplementary material for: Maternal overweight and obesity during pregnancy and sex‐dependent dysregulation of the materno‐fetal stress axis
Source: Int J Gynaecol Obstet. 2026 Jul 1;175(3):1462–87. doi: 10.1002/ijgo.71165 (PMC13769345; doi:10.1002/ijgo.71165)
Supplement: Supplementary file 1 [file IJGO-175-1462-s001.docx]

Supplement S1: Outcome details

**S1a: General and specific maternal cohort characteristics-BMI for table 1**

^1^A repeated measures ANOVA with a Greenhouse-Geisser correction determined that mean performance levels showed a statistically significant difference between measurements, F(5, 330) = 222.4, p<0.001. Bonferroni-adjusted post-hoc analysis revealed a significant difference (p <0.001) in performance between all groups, but not between second trimester vs. 6 months pp maternal weight.

^2^A repeated measures ANOVA with a Greenhouse-Geisser correction determined that mean performance levels showed a statistically significant difference between measurements, F(5, 180) = 190.2, p<0.001. Bonferroni-adjusted post-hoc analysis revealed a significant difference (p<0.001) in performance between all groups, but not between prepregnancy weight/first trimester weight vs. weight 6 months pp maternal weight.

^3^A repeated measures ANOVA with a Greenhouse-Geisser correction determined that mean performance levels showed a statistically significant difference between measurements, F(2.00, 36.0) = 45.1, p<0.001. Bonferroni-adjusted post-hoc analysis revealed a significant difference (p<0.001) in performance between all groups, but not between prepregnancy weight vs. first trimester/ 6 months pp weight and first trimester vs. 6 months pp weight maternal weight.

^4^A repeated measures ANOVA with a Greenhouse-Geisser correction determined that mean performance levels showed a statistically significant difference between measurements, F(2.67, 26.7) = 28.9, p<0.001. Bonferroni-adjusted post-hoc analysis revealed a significant difference (p<0.05) in performance between prepregnancy weight vs. second/6 months pp weight and second vs. 6 months pp and third and 5 pp maternal weight.

^5^A repeated measures ANOVA with a Greenhouse-Geisser correction determined that mean performance levels showed a statistically significant difference between measurements, F(5, 325) = 224.4, p<0.001. Bonferroni-adjusted post-hoc analysis revealed a significant difference (p<0.001) in performance between all groups, but not between first trimester vs. 6 months pp weight BMI.

^6^A repeated measures ANOVA with a Greenhouse-Geisser correction determined that mean performance levels showed a statistically significant difference between measurements, F(2.64, 92.6) = 190.3, p<0.001. Bonferroni-adjusted post-hoc analysis revealed a significant difference (p<0.001) in performance between all groups, but not between prepregnancy vs. first trimester BMI and prepregnancy/first trimester BMI vs. 6 months pp BMI.

^7^A repeated measures ANOVA with a Greenhouse-Geisser correction determined that mean performance levels showed a statistically significant difference between measurements, F(2.01, 36.3) = 47.8, p<0.001. Bonferroni-adjusted post-hoc analysis revealed a significant difference (p<0.001) in performance between all groups, but not between prepregnancy BMI vs. first trimester/ 6 months pp BMI, and first trimester BMI vs. 6 months pp BMI.

^8^A repeated measures ANOVA with a Greenhouse-Geisser correction determined that mean performance levels showed a statistically significant difference between measurements, F(2.64, 26.4) = 29.5, p<0.001. Bonferroni-adjusted post-hoc analysis revealed a significant difference (p<0.001) in performance of all groups, but not between prepregnancy BMI vs. first trimester// 6 months pp BMI and second/third trimester vs. 6 months pp weight BMI.

Sex differences are indicated in **blue for males only**, **red for females only** and **green for males and females**, and **black of sex-independent**:

^I^when analysed for sex, maternal pre-pregnancy weight was significantly different between all BMI groups in both females and males (p≤0.002) (Games-Howell post-hoc)

^II^when analysed for sex, maternal weight at first trimester was significantly different between all BMI groups in both females and males (p<0.05) (Games-Howell post-hoc)

^III^when analysed for sex, maternal weight at second trimester was significantly different between all BMI groups in both females and males (p≤0.03) (Games-Howell post-hoc)

^IV^when analysed for sex, maternal weight at third trimester was significantly different between all BMI groups in males (p<0.05), in females not between BMI 25-30 vs. BMI>30 (p=0.057) (Games-Howell post-hoc)

^V^when analysed for sex, maternal weight at birth was significantly different between all BMI groups in both females and males (p≤0.013) (Games-Howell post-hoc)

^VI^when analysed for sex, maternal weight 6 months pp was significantly different between all BMI groups in males (p≤0.001), in females between all but not between BM<25 vs. BMI 25-30 (p=0.062) (Games-Howell post-hoc)

^VII^when analysed for sex, weight retention 6mpp compared to prepregnancy weight was significantly higher in males BMI>30 vs. BMI<25 (p=0.021) (Bonferroni corrected)

^VIII^when analysed for sex, maternal prepregnancy BMI was significantly different in both females and males between BMI<25 vs. BMI 25-30 and BMI<25 vs. BMI>30 (p<0.001) (Bonferroni corrected)

^IX^when analysed for sex, maternal first trimester BMI was significantly different in both females and males between BMI<25 vs. BMI 25-30 and BMI<25 vs. BMI>30 (p≤0.005) (Bonferroni corrected)

^X^when analysed for sex, maternal second trimester BMI was significantly different in both females and males between BMI<25 vs. BMI 25-30 and BMI<25 vs. BMI>30 (p≤0.008) (Bonferroni corrected)

^XI^when analysed for sex, maternal third trimester BMI was significantly different in both females and males between BMI<25 vs. BMI 25-30 and BMI<25 vs. BMI>30 (p≤0.007) (Bonferroni corrected)

^XII^when analysed for sex, maternal BMI at birth was significantly different in both females and males between BMI<25 vs. BMI 25-30 and BMI<25 vs. BMI>30 (p<0.001) (Bonferroni corrected)

^XIII^when analysed for sex, maternal BMI 6 months pp was significantly different in both females and males between BMI<25 vs. BMI 25-30 and BMI<25 vs. BMI>30 (p<0.005) (Bonferroni corrected)

^XIV^ when analysed for sex, hair cortisone at third trimester was significantly lower in males in BMI<25 vs. BMI 25-30 (p=0.036) (Games-Howell post-hoc)

^XV^when analysed for sex, HSD11B2 protein level in peripheral placental samples was significantly lower in females in BMI>30 vs. BMI25-30 (p=0.039) (Bonferroni corrected)

**S1b: General and specific maternal cohort characteristics – GWG for table 3**

^1^A repeated measures ANOVA with a Greenhouse-Geisser correction determined that mean performance levels showed a statistically significant difference between measurements, F(2.64, 174.6) = 222.4, p<0.001. Bonferroni-adjusted post-hoc analysis revealed a significant difference (p<0.001) in performance between all groups, but not between second trimester vs. 6 months pp maternal weight.

^2^A repeated measures ANOVA with a Greenhouse-Geisser correction determined that mean performance levels showed a statistically significant difference between measurements, F(2.09, 18.8) = 15.1, p<0.001. Bonferroni-adjusted post-hoc analysis revealed a significant difference (p0<0.001) in performance between all groups, but not between prepregnancy weight/first trimester weight vs. weight 6 months pp maternal weight.

^3^A repeated measures ANOVA with a Greenhouse-Geisser correction determined that mean performance levels showed a statistically significant difference between measurements, F(2.13, 40.6) = 88.6, p0<0.001. Bonferroni-adjusted post-hoc analysis revealed a significant difference (p<0.001) in performance between all groups, but not between first trimester vs. 6 months pp weight maternal weight.

^4^A repeated measures ANOVA with a Greenhouse-Geisser correction determined that mean performance levels showed a statistically significant difference between measurements, F(2.92, 105.1) = 204.4, p<0.001. Bonferroni-adjusted post-hoc analysis revealed a significant difference (p<0.001) in performance between all groups, but not between first trimester vs. 6 months pp weight maternal weight.

^5^A repeated measures ANOVA with a Greenhouse-Geisser correction determined that mean performance levels showed a statistically significant difference between measurements, F(2.71, 176.0) = 224.4, p<0.001. Bonferroni-adjusted post-hoc analysis revealed a significant difference (p0<0.001) in performance between all groups, but not between first trimester vs. 6 months pp maternal BMI.

^6^A repeated measures ANOVA with a Greenhouse-Geisser correction determined that mean performance levels showed a statistically significant difference between measurements, F(2.02, 18.2) = 15.57, p<0.001. Bonferroni-adjusted post-hoc analysis revealed a significant difference (p<0.001) in performance between between third trimester vs. prepregnancy/first/second trimester and first trimester vs. delivery and second trimester vs. 6 months pp maternal BMI.

^7^A repeated measures ANOVA with a Greenhouse-Geisser correction determined that mean performance levels showed a statistically significant difference between measurements, F(2.19, 41.7) = 87.2, p<0.001. Bonferroni-adjusted post-hoc analysis revealed a significant difference (p<0.001) in performance between all groups, but not between prepregnancy BMI vs. first trimester/ 6 months pp maternal BMI.

^8^A repeated measures ANOVA with a Greenhouse-Geisser correction determined that mean performance levels showed a statistically significant difference between measurements, F(2.96, 103.8) = 207.1, p<0.001. Bonferroni-adjusted post-hoc analysis revealed a significant difference (p<0.001) in performance all groups, but not between first trimester vs. 6 months pp .

Sex differences are indicated in **blue for males only**, **red for females only** and **green for males and females**, and **black of sex-independent**:

^I^when analysed for sex, maternal pre-pregnancy weight in females was significantly different: recommended GWG > below GWG (p=0.013) (Bonferroni corrected)

^II^when analysed for sex, maternal weight at first trimester in females was significantly different: recommended GWG < excessive GWG (p=0.002) and below GWG < excessive GWG (p=0.029) (Bonferroni corrected)

^III^when analysed for sex, maternal weight at second trimester was significantly different: in females recommended GWG < excessive GWG (p=0.002) and below GWG < excessive GWG (p=0.010) (Bonferroni corrected)

^IV^when analysed for sex, maternal weight at third trimester was significantly different: in females recommended GWG < excessive GWG (p=0.005) and below GWG < excessive GWG (p=0.003) (Bonferroni corrected)

^V^when analysed for sex, maternal weight at at birth was significantly different: in females recommended GWG < excessive GWG (p=0.001) and below GWG < excessive GWG (p<0.001), in males recommended GWG < excessive GWG (p<0.001) and below GWG < excessive GWG (p<0.001) (Bonferroni corrected)

^VI^when analysed for sex, maternal weight 6 months pp in females was significantly different: recommended GWG < excessive GWG (p<0.001) (Bonferroni corrected)

^VII^when analysed for sex, maternal total GWG in females and males was significantly different: below GWG < recommended GWG < excessive GWG (p<0.05) (Bonferroni corrected)

^VIII^when analysed for sex, total GWG per week were significantly different in males and females (excessive GWG > recommended GWG > below GWG (p<0.005) (Bonferroni corrected)

^IX^when analysed for sex, weight retention 6m pp compared to maternal weight at birth were higher significantly in males: excessive GWG > below recommended GWG (p<0.001) (Bonferroni corrected)

^X^when analysed for sex, weight retention 6m pp compared to maternal prepregnancy weight were higher significantly in males: excessive GWG > recommended GWG (p=0.046) (Bonferroni corrected)

^XI^when analysed for sex, maternal net GWG in females and males was significantly different: below GWG < recommended GWG < excessive GWG (p<0.001) (Games Howell post-hoc)

^XII^when analysed for sex, maternal BMI prepregnancy in females was significantly different: recommended GWG < excessive GWG (p=0.010) (Bonferroni corrected)

^XIII^when analysed for sex, maternal BMI at first trimester was significantly different: in females recommended GWG < excessive GWG (p=0.001) and below recommended GWG < excessive GWG (p=0.027), in males recommended GWG < excessive GWG (p=0.019) (Bonferroni corrected)

^XIV^when analysed for sex, maternal BMI at second trimester was significantly different: in females below recommended GWG < excessive GWG (p=0.012), recommended GWG < excessive GWG (p=0.002); in males below recommended GWG < excessive GWG (p=0.033) and recommended GWG < excessive GWG (p=0.027) (Bonferroni corrected)

^XV^when analysed for sex, maternal BMI at third trimester was significantly different: in females below recommended GWG < excessive GWG (p=0.004), recommended GWG < excessive GWG (p=0.001); in males below recommended GWG < excessive GWG (p=0.027) and recommended GWG < excessive GWG (p=0.014) (Bonferroni corrected)

^XVI^when analysed for sex, maternal BMI at third trimester was significantly different: in females recommended GWG < excessive GWG (p<0.001) (Bonferroni corrected)

^XVII^when analysed for sex, maternal BMI at birth was significantly different in females: recommended GWG < excessive GWG (p<0.001) (Bonferroni corrected)

**S1c: General and specific newborn cohort characteristics – GWG for table 4**

Sex differences are indicated in **blue for males only**, **red for females only** and **green for males and females**, and **black of sex-independent**:

^I^when analysed for sex, gestational age at delivery was significantly different in males: below recommended GWG < excessive GWG (p=0.020) (Bonferroni corrected)

^II^when analysed for sex, birth weight in males was significantly different: below recommended GWG < recommended GWG (p=0.011), below recommended GWG < excessive GWG (p=0.006) (Bonferroni corrected)

^III^when analysed for sex, birth length in males was significantly different: below recommended GWG < recommended GWG (p=0.020), below recommended GWG < excessive GWG (p=0.012) (Bonferroni corrected)

^IV^when analysed for sex, birth weight to height ratio in males was significantly different: below recommended GWG < recommended GWG (p=0.020), below recommended GWG < excessive GWG (p=0.006) (Games-Howell post hoc)

^V^when analysed for sex, head circumference in males was significantly different: below recommended GWG < recommended GWG (p=0.049), below recommended GWG < excessive GWG (p=0.042) (Bonferroni corrected)
