# Supplementary material for: Maternal overweight and obesity during pregnancy and sex‐dependent dysregulation of the materno‐fetal stress axis
Source: Int J Gynaecol Obstet. 2026 Jul 1;175(3):1462–87. doi: 10.1002/ijgo.71165 (PMC13769345; doi:10.1002/ijgo.71165)
Supplement: Supplementary file 2 [file IJGO-175-1462-s002.docx]

| Maternal  characteristics | Neonatal coutcomes | | | | |
| --- | --- | --- | --- | --- | --- |
|  | Birth weight  (N) | Head circ.  (N) | Birth length  (N) | Ponderal index  (N) | Cord-blood cortisol venous  (N) |
| Maternal height | **.318**** | **.218**** | **.287^**^** | 0.012 | 0.123 |
|  | **176** | **175** | 176 | 176 | 111 |
| Maternal weight | **.187*** | 0.113 | 0.146 | 0.015 | 0.097 |
|  | **176** | 175 | 176 | 176 | 111 |
| Total GWG | **.189*** | **.151*** | **.149^*^** | 0.079 | -0.035 |
|  | **175** | **174** | 175 | 175 | 110 |
| Net GWG | 0.090 | 0.055 | 0.084 | 0.021 | -0.067 |
|  | 160 | 160 | 160 | 160 | 103 |
| Prepregnancy BMI | 0.060 | 0.041 | 0.032 | 0.005 | 0.040 |
|  | 176 | 175 | 176 | 176 | 111 |
| BMI 1^st^ trimester | 0.031 | -0.047 | -0.034 | 0.002 | -0.002 |
|  | 85 | 84 | 85 | 85 | 68 |
| BMI 2^nd^ trimester | 0.043 | -0.026 | 0.000 | -0.023 | 0.002 |
|  | 84 | 83 | 84 | 84 | 68 |
| BMI 3^rd^ trimester | -0.016 | -0.098 | -0.057 | -0.041 | 0.018 |
|  | 82 | 81 | 82 | 82 | 67 |
| BMI at birth | 0.107 | 0.099 | 0.080 | 0.012 | 0.019 |
|  | 175 | 174 | 175 | 175 | 110 |
| Total BMI gain | 0.125 | 0.107 | 0.091 | 0.078 | -0.067 |
|  | 175 | 174 | 175 | 175 | 110 |
| Cortisol 1^st^ trimester | 0.051 | 0.092 | 0.064 | -0.003 | 0.152 |
|  | 81 | 80 | 81 | 81 | 64 |
| Cortisol 2^nd^ trimester | 0.095 | 0.047 | 0.145 | 0.048 | 0.180 |
|  | 73 | 72 | 73 | 73 | 59 |
| Cortisol 3^rd^ trimester | 0.171 | 0.045 | **.259^*^** | -0.180 | 0.074 |
|  | 75 | 74 | 75 | 75 | 62 |
| Cortisol at birth | 0.110 | 0.017 | 0.107 | -0.014 | **.341**** |
|  | 155 | 155 | 155 | 155 | 102 |
| Hair cortisol 1^st^ trimester | -0.035 | 0.044 | -0.198 | 0.291 | 0.042 |
|  | 44 | 44 | 44 | 44 | 33 |
| Hair cortisol 2^nd^ trimester | 0.131 | 0.119 | 0.064 | 0.074 | -0.140 |
|  | 41 | 41 | 41 | 41 | 33 |
| Hair cortisol 3^rd^ trimester | 0.067 | 0.320 | -0.044 | 0.235 | -0.026 |
|  | 35 | 35 | 35 | 35 | 26 |
| Placenta weight | **.431**** | **.385**** | **.307^**^** | **.166*** | -0.062 |
|  | **161** | **161** | 161 | **161** | 104 |
| HSD11B2 mRNA mean | 0.190 | 0.047 | 0.186 | -0.032 | -0.205 |
|  | 66 | 66 | 66 | 66 | 52 |
| HSD11B2 mRNA peripheral | 0.086 | 0.003 | 0.053 | 0.013 | -0.084 |
|  | 66 | 66 | 66 | 66 | 52 |
| HSD11B2 mRNA central | **0.272*** | 0.083 | **0.246*** | 0.010 | -0.254 |
|  | **66** | 66 | **66** | 66 | 52 |
| HSD11B2 protein mean | -0.137 | -0.127 | -0.101 | -0.036 | -0.125 |
|  | 160 | 160 | 160 | 160 | **103** |
| HSD11B2 protein peripheral | -0.072 | -0.075 | -0.032 | -0.069 | -0.100 |
|  | 162 | 162 | 162 | 162 | 105 |
| HSD11B2 protein central | -0.127 | -0.144 | -0.142 | 0.055 | -0.057 |
|  | 166 | 166 | 166 | 166 | 109 |
| HSD11B2 %-DNA methylation: |  |  |  |  |  |
| CPG site 1 peripheral | 0.073 | 0.039 | 0.114 | -0.238 | -0.051 |
|  | 66 | 66 | 66 | 66 | 52 |
| CPG site 2 peripheral | -0.004 | 0.056 | 0.026 | -0.086 | -0.198 |
|  | 66 | 66 | 66 | 66 | 52 |
| CPG site 3 peripheral | 0.064 | 0.095 | 0.163 | -0.161 | -0.234 |
|  | 66 | 66 | 66 | 66 | 52 |
| CPG site 4 peripheral | 0.151 | 0.157 | 0.210 | -0.201 | -0.092 |
|  | 66 | 66 | 66 | 66 | 52 |
| CPG sites 1-4 peripheral | 0.127 | 0.150 | **.234^*^** | **-.252*** | -0.158 |
|  | 66 | 66 | **66** | **66** | 52 |
| CPG site 1 central | 0.225 | **.247*** | 0.212 | -0.084 | 0.115 |
|  | 66 | **66** | 66 | 66 | 52 |
| CPG site 2 central | -0.049 | 0.051 | -0.032 | -0.030 | -0.066 |
|  | 66 | 66 | 66 | 66 | 52 |
| CPG site 3 central | 0.056 | 0.169 | 0.122 | -0.127 | -0.013 |
|  | 66 | 66 | 66 | 66 | 52 |
| CPG site 4 central | 0.066 | 0.067 | 0.127 | -0.135 | -0.060 |
|  | 66 | 66 | 66 | 66 | 52 |
| CPG sites 1-4 central | 0.102 | 0.112 | 0.148 | -0.115 | 0.009 |
|  | 66 | 66 | 66 | 66 | 52 |
| CPG sites 1-4 central+peripheral | 0.111 | 0.135 | 0.201 | -0.222 | -0.044 |
|  | 66 | 66 | 66 | 66 | 52 |

Supplement S2A: Spearmen´s correlations between key maternal anthropometric and metabolic variables and neonatal outcomes. Correlation coefficients represent data from the overall analysis. independent of sex. N=number of cases; *p<0.05. **p<0.001. Sex-specific significant correlations are indicated in blue = only males: maternal height vs. birth weight k=0.319**. weight prepregnancy vs. birth weight k=0.257**. weight prepregnancy vs. birth length k=0.240*. placenta weight vs. birth weight k=0.470**. placenta weight vs. birth length k=0.409**. maternal height vs. head circ. K=0.248*. placenta weight vs. head circ. k=0.477**. mean HSD11B2 mRNA vs. birth weight k=0.540**; mean HSD11B2 mRNA vs. head circ. k=0.350*; mean HSD11B2 mRNA vs. vs. birth length k=0.352*; central HSD11B2 mRNA vs. birth weight k=0.410**; central HSD11B2 mRNA vs. birth weight k=0.529**; central HSD11B2 mRNA vs. birth length k=0.381*; peripheral HSD11B2 mRNA vs. birth weight k=0.377*; red = only females: Maternal height vs. birth weight k=0.303**. Cortisol 3^rd^ vs. birth weight k=0.446*. Cortisol 3^rd^ vs. birth length k=0.475**. placenta weight vs. birth weight k=0.386**. mean HSD11B2 mRNA vs. ponderal index k=-0.364*. CPG-1 central vs. birth weight k=0.409**. CPG-1 central vs. birth weight k=0.397*. CPG1-4 peripheral vs. body length k=0.355*. CPG-1 central vs. body length k=0.452**. CPG-1 central vs. head circ. k=0.382*. CPG-1 central vs. head circ. k=0.472*. CPG-1 peripheral vs. PI k=-0.384*. CPG-4 peripheral vs. birth length k=0.346*. CPG1-4 peripheral vs. birth length k=0.341*. CPG1-4 peripheral vs. PI k=-0.392*. CPG-1 central vs. birth length k=0.462**.CPG-2 peripheral vs. Cortisol Fv k=-0.405*. CPG-3 peripheral vs. Cortisol Fv k=-0.439*; CPG1-4 peripheral and 1-4 central vs. body length k=0.399*. HSD11B2 vs. Cortisol Fv k=-0.308*; green = males and females; black = overall analysis. independent of sex.

| Maternal  characteristics | Infant outcomes with 6 months pp | | | | |
| --- | --- | --- | --- | --- | --- |
|  | Weight  (N) | Head circumference  (N) | Height  (N) | BMI  (N) | Weight gain  (N) |
| Maternal height | 0.206 | 0.211 | **.319**** | -0.043 | 0.156 |
|  | 82 | 79 | **82** | 82 | 82 |
| Maternal weight | 0.195 | 0.098 | 0.168 | 0.069 | 0.169 |
|  | 82 | 79 | 82 | 82 | 82 |
| Total GWG | **.233*** | 0.099 | 0.087 | **.224*** | 0.213 |
|  | **82** | 79 | 82 | **82** | 82 |
| Net GWG | 0.122 | -0.029 | 0.018 | 0.156 | 0.144 |
|  | 67 | 64 | 67 | 67 | 67 |
| Prepregnancy BMI | 0.131 | 0.031 | 0.070 | 0.093 | 0.134 |
|  | 82 | 79 | 82 | 82 | 82 |
| BMI 1^st^ trimester | 0.132 | 0.020 | 0.046 | 0.120 | 0.136 |
|  | 82 | 79 | 82 | 82 | 82 |
| BMI 2^nd^ trimester | 0.155 | 0.023 | 0.059 | 0.145 | 0.162 |
|  | 81 | 78 | 81 | 81 | 81 |
| BMI 3^rd^ trimester | 0.151 | 0.027 | 0.048 | 0.162 | 0.186 |
|  | 79 | 76 | 79 | 79 | 79 |
| BMI at birth | 0.182 | 0.006 | 0.065 | 0.164 | 0.173 |
|  | 82 | 79 | 82 | 82 | 82 |
| Total BMI gain | 0.181 | 0.060 | 0.008 | **.245*** | 0.183 |
|  | 82 | 79 | 82 | **82** | 82 |
| Cortisol 1^st^ trimester | -0.043 | 0.036 | 0.006 | 0.024 | -0.084 |
|  | 78 | 75 | 78 | 78 | 78 |
| Cortisol 2^nd^ trimester | -0.102 | 0.033 | -0.105 | 0.036 | -0.188 |
|  | 71 | 68 | 71 | 71 | 71 |
| Cortisol 3^rd^ trimester | -0.071 | 0.051 | -0.043 | 0.002 | -0.091 |
|  | 72 | 69 | 72 | 72 | 72 |
| Cortisol at birth | 0.083 | 0.038 | 0.195 | -0.062 | 0.046 |
|  | 63 | 60 | 63 | 63 | 63 |
| Cortisol 5months pp | 0.057 | 0.012 | 0.027 | 0.197 | 0.007 |
|  | 53 | 50 | 53 | 53 | 53 |
| Hair cortisol 1^st^ trimester | **.357^*^** | 0.008 | **.347^*^** | 0.116 | **.390^*^** |
|  | 42 | 42 | 42 | 42 | 42 |
| Hair cortisol 2^nd^ trimester | 0.093 | -0.214 | 0.161 | -0.033 | 0.075 |
|  | 38 | 38 | 38 | 38 | 38 |
| Hair cortisol 3^rd^ trimester | 0.213 | -0.022 | -0.022 | 0.232 | 0.178 |
|  | 32 | 32 | 32 | 32 | 32 |
| Placenta weight | **.311*** | **.345**** | **.301*** | 0.148 | -0.017 |
|  | **67** | **64** | **67** | 67 | 67 |
| HSD11B2 mRNA mean | 0.092 | -0.233 | 0.184 | 0.010 | 0.057 |
|  | 43 | 41 | 43 | 43 | 43 |
| HSD11B2 mRNA peripheral | 0.049 | -0.281 | 0.092 | -0.048 | 0.064 |
|  | 43 | 41 | 43 | 43 | 43 |
| HSD11B2 mRNA central | 0.061 | -0.139 | 0.132 | 0.041 | -0.040 |
|  | 43 | 41 | 43 | 43 | 43 |
| HSD11B2 protein mean | -0.221 | -0.074 | -0.142 | -0.183 | -0.156 |
|  | 67 | 65 | 67 | 67 | 67 |
| HSD11B2 protein peripheral | **-0.238*** | -0.090 | -0.015 | **-0.274*** | -0.196 |
|  | **69** | 66 | 69 | **69** | 69 |
| HSD11B2 protein central | -0.001 | -0.113 | -0.104 | 0.050 | 0.004 |
|  | 72 | 70 | 72 | 72 | 72 |
| HSD11B2 %-DNA methylation: |  |  |  |  |  |
| CPG site 1 peripheral | -0.035 | -0.121 | 0.035 | -0.054 | -0.001 |
|  | 43 | 41 | 43 | 43 | 43 |
| CPG site 2 peripheral | 0.047 | **.309*** | 0.118 | -0.058 | -0.034 |
|  | 43 | **41** | 43 | 43 | 43 |
| CPG site 3 peripheral | 0.143 | 0.245 | 0.178 | 0.007 | 0.079 |
|  | 43 | 41 | 43 | 43 | 43 |
| CPG site 4 peripheral | 0.005 | -0.131 | 0.118 | -0.071 | -0.089 |
|  | 43 | 41 | 43 | 43 | 43 |
| CPG sites 1-4 peripheral | 0.088 | 0.141 | 0.209 | -0.046 | -0.005 |
|  | 43 | 41 | 43 | 43 | 43 |
| CPG site 1 central | 0.137 | -0.097 | **.325*** | -0.039 | 0.107 |
|  | 43 | 41 | **43** | 43 | 43 |
| CPG site 2 central | 0.033 | 0.113 | 0.145 | -0.061 | 0.007 |
|  | 43 | 41 | 43 | 43 | 43 |
| CPG site 3 central | 0.133 | 0.168 | 0.282 | -0.009 | 0.049 |
|  | 43 | 41 | 43 | 43 | 43 |
| CPG site 4 central | -0.075 | -0.205 | -0.011 | -0.038 | -0.066 |
|  | 43 | 41 | 43 | 43 | 43 |
| CPG sites 1-4 central | 0.051 | -0.128 | 0.190 | -0.051 | 0.004 |
|  | 43 | 41 | 43 | 43 | 43 |
| CPG sites 1-4 central+peripheral | 0.038 | -0.042 | 0.192 | -0.085 | -0.027 |
|  | 43 | 41 | 43 | 43 | 43 |

Supplement S2B: Spearmen´s correlations between key maternal anthropometric and metabolic variables and infant outcomes 6 month post partum. Correlation coefficients represent data from the overall analysis. independent of sex. N=number of cases; *p<0.05. **p<0.001. Sex-specific significant correlations are indicated in blue = only males: Placenta weight vs. body weight 6 months pp k=0.411*. Maternal height vs. height 6 months pp k=0.327*. hair cortisol 1^st^ trimester vs. weight 6 months pp k=0.589**. hair cortisol 1^st^ trimester vs. height 6 months pp k=0.516*. hair cortisol 1^st^ trimester vs. weight gain 6 months pp k=0.521**. placenta weight vs. height 6 months pp k=0.328*. mean HSD11B2 mRNA vs. height 6 months pp k=0.436*. CPG-2 central vs. head circ. 6 months pp k=-0.415*. CPG1-4 central vs. head circ. 6 months pp k=-0.479*. BMI 2^nd^ vs. BMI 6 months pp k= 0.300*. Cortisol 2^nd^ vs. weight gain 6 months pp k= -0.353*; red = only females: mean HSD11B2 mRNA vs. height 6 months pp k=-0.558**. CPG-1 central vs. weight 6 months pp k=0.513*. CPG-3 central vs. weight 6 months pp k=0.527*. CPG1-4 central vs. weight 6 months pp k=0.459*. CPG-3 peripheral vs. height 6 months pp k=0.479*. CPG-1-4 peripheral vs. height 6 months pp k=0.517*. CPG-1 central vs. height 6 months pp k=0.610**. CPG-3 central vs. height 6 months pp k=0.670**. CPG1-4 central vs. height 6 months pp k=0.514*. CPG-2 peripheral vs. head circ. 6 months pp k= 0.453*. CPG-3 peripheral vs. head circ. 6 months pp k= 0.453*. CPG-2 central vs. head circ. 6 months pp k= 0.509*. CPG-3 vs. head circ. 6 months pp k= 0.502*. CPG sites 1-4 central+peripheral vs. height 6 months pp k=0.501*; green = males and females; black = overall analysis. independent of sex.

| Neonatal  characteristics | Infant outcomes with 6 months pp | | | | |
| --- | --- | --- | --- | --- | --- |
|  | Weight  (N) | Head circumference  (N) | Height  (N) | BMI  (N) | Weight Gain  (N) |
| Birth weight (g) | **.508**** | **.374**** | **.442**** | **.292**** | 0.045 |
|  | **82** | **79** | **82** | **82** | 82 |
| Height (cm) | **.415**** | **.353**** | **.368**** | **.240*** | 0.110 |
|  | **82** | **79** | **82** | **82** | 82 |
| Head circumference (cm) | **.448**** | **.522**** | **.462**** | **.234*** | 0.155 |
|  | **81** | **78** | **81** | **81** | 81 |
| PI at birth | 0.098 | 0.022 | 0.054 | 0.077 | -0.154 |
|  | 82 | 79 | 82 | 82 | 82 |
| Cortisol fetal venous | -0.023 | -0.114 | 0.068 | -0.148 | -0.041 |
|  | 66 | 63 | 66 | 66 | 66 |

Supplement S2C: Spearmen´s correlations between key neonatal anthropometric and metabolic variables and infant outcomes 6 month post partum. Correlation coefficients represent data from the overall analysis. independent of sex. N=number of cases; *p<0.05. **p<0.001. Sex-specific significant correlations are indicated in blue = only males: birth weight vs. weight 6 months pp k=0.487**. birth length vs. height 6 months pp k= 0.380**. birth weight vs. height 6 months pp k=0.437**. head circ. at birth vs. height 6 months pp k=0.383**. PI birth vs. height 6 months pp k=0.307*; red = only females: birth weight vs. weight 6 months pp k=0.481**. birth length vs. weight 6 months pp k=0.410*. head circ. at birth vs. weight 6 months pp k=0.408*. birth weight vs. height 6 months pp k=0.386*. birth length vs. height 6 months pp k=0.542**. head circ. vs. height 6 months pp k=0.516**. birth weight vs. head circ. 6 months pp k=0.393*. head circ. vs. head circ. 6 months pp k=0.597**. PI at birth vs. BMI 6 months pp k=0.347*; green = males and females; black = overall analysis. independent of sex.
